# Vibrational Study on Structure and Bioactivity of Protein Fibers Grafted with Phosphorylated Methacrylates

**DOI:** 10.3390/molecules26216487

**Published:** 2021-10-27

**Authors:** Michele Di Foggia, Masuhiro Tsukada, Paola Taddei

**Affiliations:** 1 Department of Biomedical and Neuromotor Sciences, University of Bologna, via Belmeloro 8/2, 40126 Bologna, Italy; michele.difoggia2@unibo.it; 2 Division of Applied Biology, Faculty of Textile Science and Technology, Shinshu University, Ueda 386-8567, Japan; tsukada@shinshu-u.ac.jp

**Keywords:** silk fibroin, *Bombyx mori*, Tussah, wool keratin, grafting, phosmer, Raman spectroscopy, IR spectroscopy

## Abstract

In the last decades, silk fibroin and wool keratin have been considered functional materials for biomedical applications. In this study, fabrics containing silk fibers from *Bombyx mori* and Tussah silk fibers from *Antheraea pernyi*, as well as wool keratin fabrics, were grafted with phosmer CL and phosmer M (commercial names, i.e., methacrylate monomers containing phosphate groups in the molecular side chain) with different weight gains. Both phosmers were recently proposed as flame retarding agents, and their chemical composition suggested a possible application in bone tissue engineering. IR and Raman spectroscopy were used to disclose the possible structural changes induced by grafting and identify the most reactive amino acids towards the phosmers. The same techniques were used to investigate the nucleation of a calcium phosphate phase on the surface of the samples (i.e., bioactivity) after ageing in simulated body fluid (SBF). The phosmers were found to polymerize onto the biopolymers efficiently, and tyrosine and serine underwent phosphorylation (monitored through the strengthening of the Raman band at 1600 cm^−1^ and the weakening of the Raman band at 1400 cm^−1^, respectively). In grafted wool keratin, cysteic acid and other oxidation products of disulphide bridges were detected together with sulphated residues. Only slight conformational changes were observed upon grafting, generally towards an enrichment in ordered domains, suggesting that the amorphous regions were more prone to react (and, sometimes, degrade). All samples were shown to be bioactive, with a weight gain of up to 8%. The most bioactive samples contained the highest phosmers amounts, i.e., the highest amounts of phosphate nucleating sites. The sulphate/sulphonate groups present in grafted wool samples appeared to increase bioactivity, as shown by the five-fold increase of the IR phosphate band at 1040 cm^−1^.

## 1. Introduction

Thanks to their excellent properties (environmental stability, remarkable mechanical properties, controlled proteolytic biodegradability, morphologic flexibility, biocompatibility), silk fibroin and wool keratin are outstanding proteinaceous materials that have found numerous applications in the biomedical field [1,2,3]; the possibility of using these materials for tissue engineering is a subject of broad interest.

Their outstanding features derive from their characteristic structure. Silk is composed of a core containing at least two main water-insoluble fibroin proteins (light and heavy chains), coated by a water-soluble protein, i.e., sericin. Silk is produced from insects or domestic (i.e., *Bombyx mori*) and wild silkworm (i.e., *Antheraea pernyi* (Tussah), *Philosamia c. ricini*, etc.) species, with different structural motifs and amino acid compositions.

The most abundant amino acid in *B. mori* silk fibroin is glycine (Gly, 42.9 mol%), followed by alanine (Ala, 30.0 mol%), serine (Ser, 12.2 mol%), tyrosine (Tyr, 4.8 mol%), and valine (Val, 2.5 mol%) [4]. In the fibroin heavy chain, two primary regions, described as ‘crystalline’ and ‘amorphous’, regularly alternate [5]. Twelve repeating sequences were identified along the fibroin heavy chain, comprising various repeating units, such as the highly repetitive AGSGAG hexamer, which forms the crystalline regions (anti-parallel β-sheet conformation), and the relatively less repetitive AGYGAG and/or AGVGYGAG motifs, which form the semi-crystalline regions. The twelve repeating regions are connected by eleven nonrepetitive amorphous regions that contain negatively charged, polar, bulky hydrophilic and aromatic amino acids.

The amino acid composition of Tussah silk fiber in the crystalline region is characterized by a large amount of alanine (Ala 48.6 mol%) [6], and –(Ala)_n_– sequences are reported to form the primary structure (anti-parallel β-sheet conformation) [7]. Ala-rich regions alternate with glycine-rich regions (Gly 28.3 mol%), which contain a variety of other amino acids such as serine (Ser 8.9 mol%), tyrosine (Tyr 3.7 mol%), aspartic acid (Asp 3.9 mol%), arginine (Arg 2.6 mol%) and tryptophan (Trp 0.5 mol%) [6].

Compared with silk, wool fiber is a lower crystallinity composite material comprising cuticle and cortical cells as building blocks [8]. Cuticle cells, forming the most external layer of the fiber, are composed of keratin in β-sheet and disordered conformations, while the more internal cortical cells, forming the fiber core, are constituted by keratin in α-helix conformation. Compared to cortical cells, cuticle cells have a higher proportion of cysteine, proline, serine and valine and lower amounts of arginine, aspartic and glutamic acids, phenylalanine, threonine and leucine [9].

Many strategies have been employed to functionalize these natural polymers to improve their properties according to the desired function, to create high-performing materials for traditional and new applications. Among them, graft copolymerisation techniques using vinyl monomers were introduced in Japan in the early 1960s and have been widely applied in the textile industry [10]. The grafting monomers such as methacrylamide (MAA) [11] and 2-hydroxyethyl methacrylate (HEMA) [12] and a grafting binary system containing MAA and HEMA [13,14] were successfully applied on the industrial scale for silk processing. The graft copolymerisation techniques use grafting agents that modify the silk properties depending on the molecular weight and functional groups of the grafted polymer and the grafting extent [13,15,16].

More recently, the possibility of producing flame retardant silk fabrics has been explored by grafting the biopolymer with vinyl monomers containing phosphate groups in the side chain [17,18], two additives in particular, i.e., phosmer M (2-phosphonooxyethyl 2-methylprop-2-enoate) and phosmer CL ((3-chloro-2-phosphonooxypropyl). 2-methylprop-2-enoate) proved particularly useful to achieve better flame retardancy in silk fibroin from *Bombyx mori* [18]. Preliminary results showed that grafting increased the thermal stability of the fibers without negatively affecting mechanical properties. SEM images showed that the surface of the grafted silk fibers was as smooth and flat as that of control silk fibers. It appeared that flame retardant silk fabrics with good performance could be prepared by limiting the weight gain in the range of 5–25%, which is the safest for keeping the intrinsic silk fabric properties unchanged.

In this study, proteins fibers, including silk fibroin from *Bombyx mori*, silk fibroin from *Antheraea pernyi* (Tussah) and wool keratin in the form of fabrics, were grafted with phosmer CL and phosmer M with different weight gains. The structure of the grafted samples was investigated using Raman and IR spectroscopy to determine possible changes induced by the grafting and identify the most reactive amino acids towards the phosphorylated methacrylate monomers.

On the other hand, the chemical composition of the phosmers (i.e., the presence of phosphate groups in the side chain) suggests that they may act as nucleating sites for calcium phosphates/apatite deposition. Actually, the introduction of negatively charged groups such as phosphates [19,20], carboxylates [21], sulphonates [22] and anionic polypeptides [23] proved an excellent strategy to enhance apatite formation. Phosphate groups are known to play a key role in mineralization processes in vivo and in vitro [24,25,26].

In this context, in view of the possible applications of phosmer-grafted substrates in bone tissue engineering, the bioactivity of the fabrics was studied in vitro, upon ageing at 37 °C in a simulated body fluid (SBF), having ion concentrations nearly equal to those of human blood plasma. Since 1990 [27], this has been a commonly accepted method to test the apatite forming ability of materials. Vibrational spectroscopy was used to investigate the possible formation of calcium phosphate phases on the surface of the samples.

## 2. Results

Table 1 shows the analyzed samples, hereinafter identified through their % weight gains. A previous study showed that the sample weight gain toward protein fibers using phosmer CL was higher than using phosmer M, probably due to the synergistic effect of the chlorine atom in phosmer CL [18].

### 2.1. Vibrational Analyses of Grafted B. mori Silk Fibroin Fabrics

**Figure 1:**
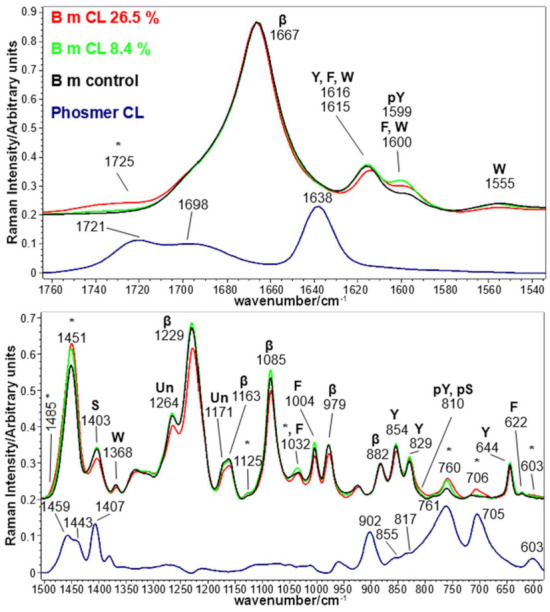
Raman spectra of *B. mori* silk fibroin fabrics before (control) and after grafting with phosmer CL (weight gains 8.4% and 26.5%). The spectra are normalized to the Amide I band (1667 cm^−1^). The spectrum of phosmer CL is reported for comparison; the main bands that in the grafted samples strengthened due to its contribution are indicated with * (see Supplementary Material for assignments). The main bands assignable to β-sheet (β) or unordered (Un) conformation as well as to tyrosine (Y), phosphorylated tyrosine (pY), phenylalanine (F), serine (S), phosphorylated serine (pS) and tryptophan (W) are indicated.

**Figure 2:**
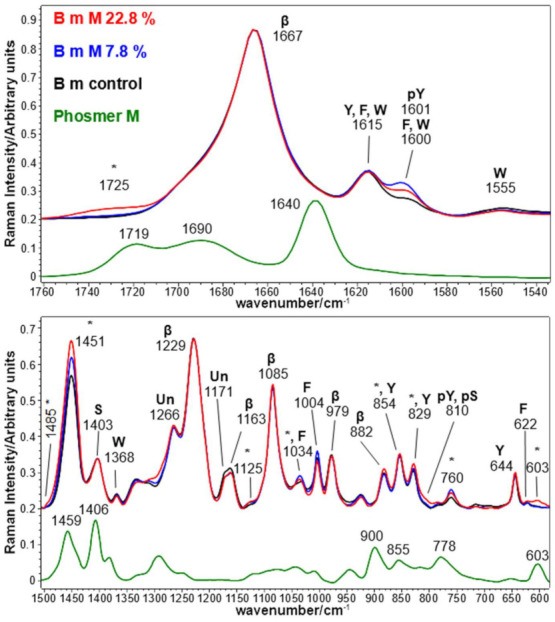
Raman spectra of *B. mori* silk fibroin fabrics before (control) and after grafting with phosmer M (weight gains 7.8% and 22.8%). The spectra are normalized to the Amide I band (1667 cm^−1^). The spectrum of phosmer M is reported for comparison; the main bands that in the grafted samples strengthened due to its contribution are indicated with * (see Supplementary Material for assignments). The main bands assignable to β-sheet (β) or unordered (Un) conformation as well as to tyrosine (Y), phosphorylated tyrosine (pY), phenylalanine (F), serine (S), phosphorylated serine (pS) and tryptophan (W) are indicated.

Figure 1 and Figure 2 show the Raman spectra of silk fabrics grafted with phosmer CL and phosmer M, respectively. The Raman spectra of control silk fabric and phosmers are reported for comparison. Band wavenumbers and assignments are reported in Table S1, Supplementary Material.

The main bands ascribable to phosmers (marked with asterisks) were observed at about 1725, 760, 603 and 706 cm^−1^ (the latter only for phosmer CL, see Table S1, Supplementary Material for assignments [28,29,30,31,32]). Interestingly, in the grafted samples, the ester C=O stretching mode at about 1725 cm^−1^ appeared shifted with respect to pure phosmers, suggesting the involvement of this group in a different pattern of hydrogen bonds.

Other bands were found to strengthen upon grafting, such as the CH_2_ bending mode at about 1450 cm^−1^, which also showed an increase of its full-width at half-maximum (FWHM) from 25 cm^−1^ (control) to 28 cm^−1^ (grafted samples). Moreover, a new component was detected at 1485 cm^−1^, in agreement with the trend observed upon grafting with HEMA [33].

**Figure 3:**
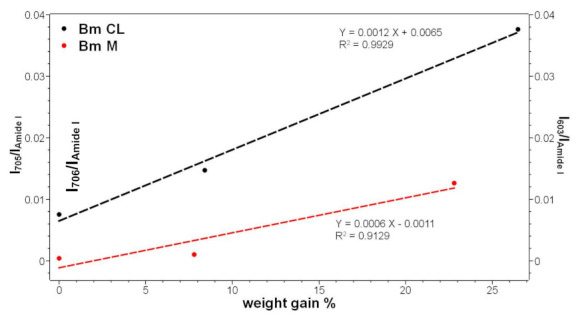
Trend of the I_706_/I_Amide I_ (black) and I_603_/I_Amide I_ (red) Raman intensity ratios as a function of the weight gain for the silk fibroin fabrics grafted with phosmer CL (black) and phosmer M (red), respectively.

The above-mentioned bands showed increased intensity at increased weight gain. In particular, the Raman I_706_/I_Amide I_ and I_603_/I_Amide I_ intensity ratios could be used as spectral markers to obtain quantitative information on phosmer CL and phosmer M incorporation (Figure 3). Both ratios well correlated with the sample weight gain as well as I_1725_/I_Amide I_ and I_1450_/I_Amide I_ (Figure S1, Supplementary Material).

In the CC stretching range [31,32], slight enhancements were detected for the bands at about 1125 and 1035 cm^−1^; these bands were observed to strengthen also upon grafting with HEMA [33], being characteristic of poly HEMA (i.e., they appeared as distinct spectral features upon polymerization of HEMA). Their strengthening suggests that phosmers polymerize onto silk fibroin; actually, both phosmers show broad and weak bands in this spectral region. This interpretation is confirmed by the disappearance of the bands specifically assignable to the C=CH_2_ group (see discussion below).

The main phosphate band, reported at about 1080 cm^−1^ [34,35,36], was observed as a weak spectral feature also in pure phosmers, and the grafted samples would overlap the strong silk fibroin band at 1085 cm^−1^.

The incorporation of phosmers may have caused structural changes into silk fibroin conformation. Raman spectra showed that all samples maintained the overall β-sheet structure, as shown by the position of Amide I and III (at 1667 and 1229 cm^−1^ [29,30,37], respectively) as well as by the other bands sensitive to this structure, i.e., at 1163, 1085, 979 and 882 cm^−1^ (see Table S1, Supplementary Material for assignments [29,30,37]).

The FWHM of the Raman Amide I band was nearly the same in the spectra of all the fabrics. Therefore, as expected, the Amide I band fitting analysis did not disclose any variation of secondary structure distribution: in all the examined fabrics, the main conformation remained β-sheet (about 56%) [38]. Both phosmers have some intense Raman bands that overlap the Amide I spectral region, i.e., at 1698 and 1638 cm^−1^ (phosmer CL) and 1690 and 1640 cm^−1^ (phosmer M); in the spectra of the grafted samples, as previously observed, a phosmer band at about 1725 cm^−1^ appeared. On the other hand, no similar strengthening was detected near 1640 cm^−1^, where olefinic C=C stretching falls, suggesting that no residual monomer remained trapped in the grafted fabrics nor detectable amounts of C=C bonds remained unreacted due to polymerization. In confirmation, as expected, the phosmer band at about 900 cm^−1^ was never detected in the Raman spectra of the grafted fabrics since it is assignable to =CH_2_ wagging [39]; analogously, in the latter spectra, any contribution from the phosmers band at about 1400 cm^−1^ can be excluded, being attributable to =CH_2_ deformation.

**Figure 4:**
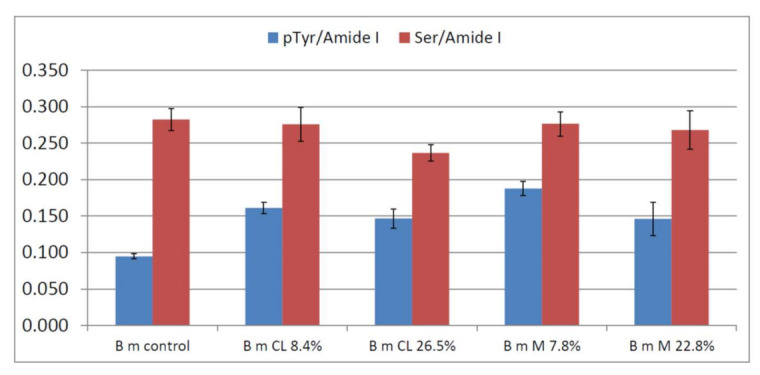
Values of the I_pTyr_/I_Amide I_ and I_Ser_/I_Amide I_ intensity ratios (average ± standard deviation) obtained from the Raman spectra of *B. mori* silk fibroin fabrics under study.

The C=C stretching modes of aromatic amino acids (mainly Tyr, Phe and Trp) at about 1620 and 1600 cm^−1^ exhibited interesting variations after grafting. In fact, the band at 1616 cm^−1^ remained almost constant in intensity and position (except for the 26.5% CL grafted sample, see below), while the band at 1600 cm^−1^ increased in intensity in all the grafted fabrics (see below, Figure 4 for more quantitative results). According to Zhang et al. [36], this trend may be ascribed to the phosphorylation of Tyr residues’ phenolic -OH group. The reaction scheme between phosmers and the Tyr side chain of silk fibroin is shown in Figure S2, Supplementary Material. Interestingly, the intensity of the band at 1600 cm^−1^ followed the trends CL 8.4% > CL 26.5% > control for CL grafting and M 7.8% > M 22.8% > control for M grafting.

In other words, the grafted silk fabrics with the highest weight gains (i.e., CL 26.5% and M 22.8%) showed a 1600 cm^−1^ band intensity reduction compared to those with the lowest weight gains. In the CL 26.5% grafted sample, this trend may be related to the weakening of several spectral features ascribable to Tyr upon grafting: this is the case of the bands at 1616, 854, 829 and 644 cm^−1^, which in the CL 26.5% grafted fabric showed a significantly lower intensity than in the control sample. The same behavior was observed for other bands assigned to aromatic amino acids, i.e., at 1555 and 1368 cm^−1^ (Trp) and 1004 cm^−1^ (Phe). This trend confirmed a preferential degradation of amorphous domains containing bulky aromatic residues [40], with loss of amino acids such as Tyr residues which became unavailable to react. On the other hand, the weakening of the 1403 cm^−1^ band in the CL 26.5% sample may be ascribed to the involvement of Ser residues in grafting with phosmers. Ashton et al. [41] have reported a reduction in the intensity of this band upon phosphorylation of Ser residues. According to the assignments reported by Ashton et al. [41], the involvement of Ser and Tyr in phosphorylation and thus in grafting would be confirmed by the shoulder at 810 cm^−1^, particularly evident in both CL 26.5% and M 22.8% samples.

To gain more insights into the reactivity of Tyr and Ser residues, useful marker Raman intensity ratios were identified, i.e., I_pTyr_/I_Amide I_ (between the band at about 1600 cm^−1^ and Amide I) and I_Ser_/I_Amide I_ (between the band at about 1400 cm^−1^ and Amide I), and their trends are reported in Figure 4. As can be easily seen, I_pTyr_/I_Amide I_ showed more significant changes than I_Ser_/I_Amide I_. The former ratio increased with weight gain at low values of this parameter; the samples with the highest weight gains showed decreased ratio due to fibroin degradation and loss of Tyr residues.

According to the literature, the I_854_/I_829_ intensity ratio trend was evaluated to gain information on the Tyr environment [42]. This intensity ratio has been used to estimate the state of hydrogen bonding involving the Tyr OH group. If the Tyr residue is buried, the OH group acts as a strong H-bond donor (i.e., with a COO^−^ group), and the intensity ratio achieves its minimum value (about 0.3) [42]. When the Tyr residue is exposed to an aqueous solution, the OH group acts both as a donor and an acceptor of moderate/weak strength H-bonds, and the intensity ratio is approximately 1.25 [42]. When the phenoxyl oxygen accepts a strong H-bond from an electropositive group (such as an NH_3_^+^ group) and does not participate in significant H-bond donation, the intensity ratio approaches the presumed maximum value of 2.5 [42]. In CL grafted samples, the I_854_/I_829_ intensity ratio reduced from 1.40 (control) to 1.2 (CL 26.5%), indicating a lower exposure of Tyr residues to the silk surface, possibly due to the reaction with phosmer. Similar behavior was also detected in M grafted fabrics, but in this case, it must be recalled that the phosmer may contribute to the intensity of both the 854 and 829 cm^−1^ bands.

**Figure 5:**
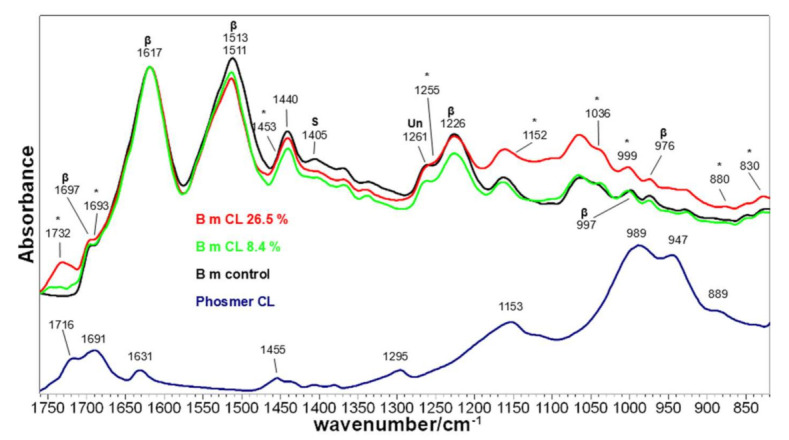
IR spectra of *B. mori* silk fibroin fabrics before (control) and after grafting with phosmer CL (weight gains 8.4% and 26.5%). The spectra are normalized to the Amide I band at 1617 cm^−1^. The spectrum of phosmer CL is reported for comparison. Asterisks (*) indicate the main spectral features ascribable to phosmer (see Supplementary Material for assignments). The main bands assignable to β-sheet conformation (β), unordered (Un) structures and serine (S) are indicated.

**Figure 6:**
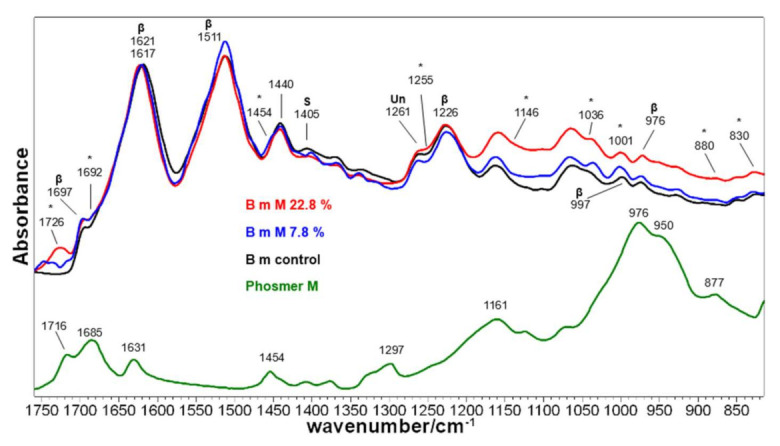
IR spectra of *B. mori* silk fibroin fabrics before (control) and after grafting with phosmer M (weight gains 7.8% and 22.8%). The spectra are normalized to the Amide I band at 1617 cm^−1^. The spectrum of phosmer M is reported for comparison. Asterisks (*) indicate the main spectral features ascribable to phosmer (see Supplementary Material for assignments). The main bands assignable to β-sheet conformation (β), unordered (Un) structures and serine (S) are indicated.

Figure 5 and Figure 6 show the IR spectra of silk fabrics grafted with phosmers CL and M, respectively. Spectra of control silk fabrics and phosmers are reported for comparison. Band wavenumbers and assignments are reported in Table S2, Supplementary Material.

The main bands ascribable to phosmers were observed at about 1730‒1690, 1150, 1036 and 1000 cm^−1^; minor bands at about 1450, 880 and 830 cm^−1^ were detected (see Table S2, Supplementary Material for assignments [17,31,32,43]). They showed a generally increased intensity at increased weight gain and appeared often shifted with respect to pure phosmers, as above observed. Moreover, the spectral background in the 1300–650 cm^−1^ range appeared progressively higher due to the contribution of the broad and strong band of phosmers in this spectral region.

**Figure 7:**
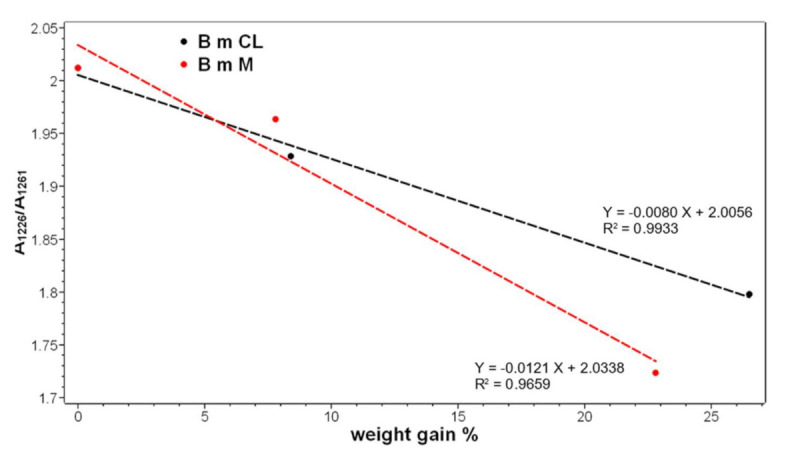
Trend of the A_1226_/A_1261_ IR absorbance ratio as a function of the weight gain for the silk fibroin fabrics grafted with phosmer CL (black) and phosmer M (red).

IR spectra confirmed that the overall conformation of the grafted fabrics remained β-sheet, as evidenced by the presence of the typical bands of this structure at about 1697 and 1621–1617 cm^−1^ (Amide I), 1513–1511 cm^−1^ (Amide II), 1226 cm^−1^ (Amide III) and 997 and 976 cm^−1^ (Ala-Gly sequences) [44,45]. Upon grafting with phosmer M, a slight wavenumber shift was observed for the Amide I band, i.e., from 1617 cm^−1^ (control) to 1621 cm^−1^ (M 22.8%). Interestingly, while the FWHM of the IR Amide I remained almost unchanged in all samples (thus confirming Raman results), the Amide II band reduced its FWHM upon grafting (from 52 to 50 cm^−1^ in CL samples and to 48 cm^−1^ in M samples). All these spectral features suggest a certain structural rearrangement. This finding could be related to the change of the IR A_1226_/A_1261_ absorbance ratio, which is considered a marker of the degree of crystallinity of silk fibroin [40], i.e., of the β-sheet content. Actually, the Amide III component at 1226 cm^−1^ is due to the latter conformation, while the band at 1261 cm^−1^ is assignable to unordered structures [29] (Table S2, Supplementary Material). As mentioned in the Introduction, crystalline regions of silk fibroin have a prevailing β-sheet conformation and are rich in simple amino acids (Ala, Gly, Ser), while amorphous regions are rich in bulky and polar amino acids [40]. In agreement with previous studies [33], the A_1226_/A_1261_ ratio was found to decrease upon grafting, suggesting that the grafted polymer introduced a certain disorder into the fiber; as shown in Figure 7, the ratio well correlates to weight gain for both phosmers. However, it must be observed that A_1226_/A_1261_ may also change because of the contribution of the band at about 1255 cm^−1^, which is assignable to P=O stretching of the phosphate group [17,43].

It is not surprising that structural rearrangements were observed only in IR spectra and not in the Raman ones. Actually, the latter represents the sample bulk (which evidently did not undergo any change). At the same time, the ATR technique allowed information to be gained on the surface skin of the fabrics, since, under the used experimental conditions (i.e., using a diamond crystal), the penetration depth of the IR radiation (and thus the sampling depth) is 2 μm. It is evident that grafting modified, although slightly, only the surface of the fibers. On the other hand, in a previous study [18], it has been reported that grafting occurs inside the fiber. In any case, the detection of the phosmers indicates that they are located within the first 2 μm of the fibers at least.

IR spectroscopy confirmed Ser residues’ involvement in the grafting reaction: both CL and M grafted samples showed a decrease in intensity of the band at 1405 cm^−1^, assignable to Ser [46,47]. A similar trend was observed upon sulphation [46,47].

### 2.2. Vibrational Analyses of Grafted Tussah Silk Fibroin Fabrics

**Figure 8:**
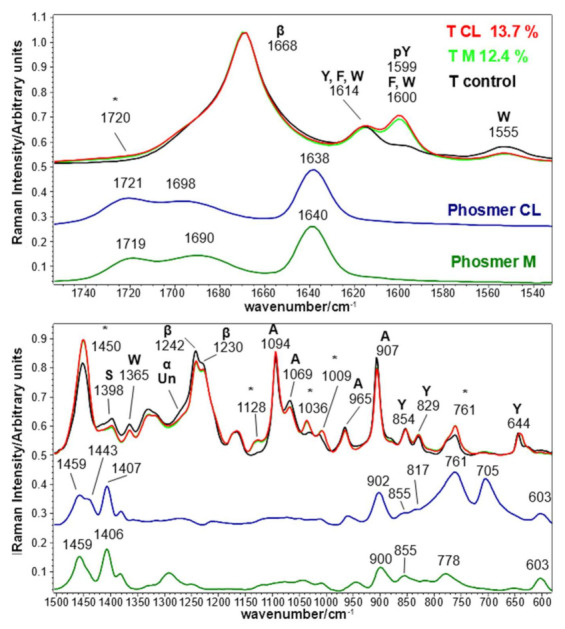
Raman spectra of Tussah silk fibroin fabrics before (control) and after grafting with phosmer CL (weight gain 13.7%) and M (weight gain 12.4%). The spectra are normalized to the Amide I band. The spectra of phosmers CL and M are reported for comparison; the main bands that in the grafted samples strengthened due to their contribution are indicated with * (see Supplementary Material for assignments). The main bands assignable to β-sheet (β), α-helix (α) or unordered (Un) conformation as well as to tyrosine (Y), phenylalanine (F), phosphorylated tyrosine (pY), serine (S), tryptophan (W) and poly-alanine-rich segment (A) are indicated.

The Raman spectra of Tussah silk fabrics grafted with phosmers are shown in Figure 8. Band wavenumbers and assignments are reported in Table S3, Supplementary Material.

Raman spectra are dominated by the bands sensitive to β-sheet conformation: Amide I at 1668 cm^−1^, Amide III at about 1242–1230 cm^−1^ and the bands at 1094, 1069, 965, and 907 cm^−1^ assignable to alanine sequential segments adopting the same structure [48,49,50]. The spectra of the grafted fabrics were also characterized by several spectral features assignable to phosmers (marked with asterisks) or to the grafting mechanism, as previously described: they were observed at about 1720, 1450, 1128, 1036, 1009 and 761 cm^−1^ (Figure 8, see Table S3, Supplementary Material for assignments). The detection of the bands at 1128 and 1036 cm^−1^ suggest, as observed for *B. mori* fabrics, that the phosmers effectively polymerized onto Tussah silk. The structure was slightly affected by grafting (more than in *B. mori* fabrics): a slight reduction of the Amide I FWHM from 23 to 22 cm^−1^ was measured after grafting, indicating a more regular structure in grafted samples. Curve fitting of the Amide I band confirmed the previous finding since the β-sheet content increased from 60% (control) to 63% in grafted fabrics with a decrease of α-helix (from 14% to 13%) and unordered structures (from 11% to 9%). Accordingly, the Amide III profile showed a weakening of the broad shoulder between 1260 and 1270 cm^−1^ (α-helix and unordered structures). The Raman spectra showed that grafting induced changes in the Tyr environment: similarly to *B. mori* fabrics, the I_854_/I_829_ intensity ratio decreased from 1.38 (control) to 1.25 for grafted fabric, thus confirming a reduced exposure of Tyr residues induced by the treatment. The strengthening of the band at 1599 cm^−1^ upon grafting indicates the phosphorylation of Tyr residues, as previously described in *B. mori* fabrics. Among the Trp spectral features, the band at 1555 cm^−1^ weakened, while the band at 760 cm^−1^ showed an increased intensity due to the contribution of grafted phosmers. Analogous behavior was observed above for *B. mori* silk fibroin fabrics. Trp residues have been reported to be preferentially located in unordered domains, which are more prone to proteolytic degradation [51]. In this light, it is not surprising that Raman spectra showed enrichment in ordered conformations due to a partial loss of amino acids in the amorphous regions. Differently from silk fibroin at the highest weight gains, the relative intensity of the Tyr bands did not appear to decrease.

The involvement of Ser residues in grafting is evidenced by the decrease in intensity of the 1398 cm^−1^ band, already observed in *B. mori* silk fibroin fabrics. In this case, Ser loss under grafting conditions should be considered negligible, since in Tussah silk fibers, Ser residues are present preferentially in ordered conformations (60% in β-sheet and 20% in α-helix) [51].

**Figure 9:**
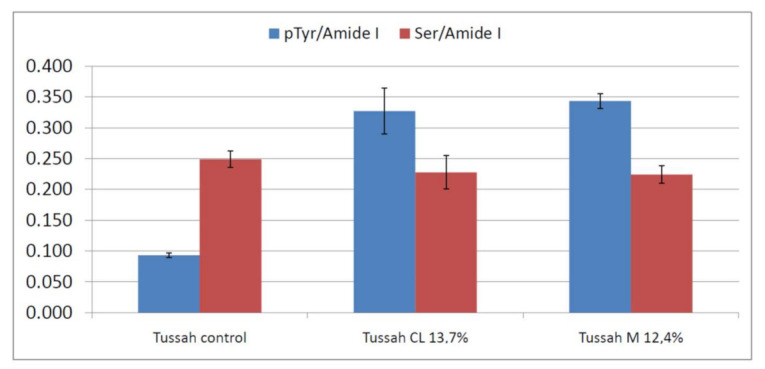
I_pTyr_/I_Amide I_ and I_Ser_/I_Amide I_ intensity ratios (average ± standard deviation, n = 4) obtained from the Raman spectra of Tussah silk fibroin fabrics under study.

Figure 9 reports the trend of the I_pTyr_/I_Amide I_ and I_Ser_/I_Amide I_ intensity ratios, used to gain information on the reactivity of Tyr and Ser residues upon grafting. As can be easily seen, both ratios significantly changed upon grafting; the former increased and the latter decreased due to Tyr and Ser phosphorylation, respectively. The changes appeared more significant than B m CL 8.4%, M 7.8% and M 9.6% *B. mori* silk fibroin fabrics, as expected based on the lower weight gains of the latter samples.

**Figure 10:**
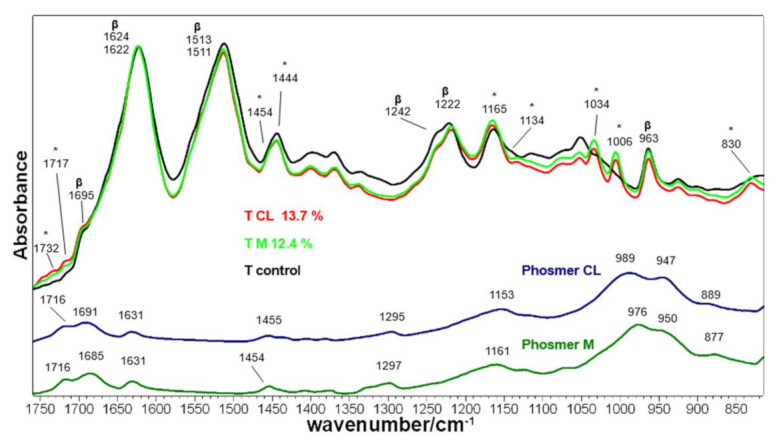
IR spectra of Tussah silk fibroin fabrics before (control) and after grafting with phosmer CL (weight gain 13.7%) and M (weight gain 12.4%). The spectra are normalized to the Amide I band. The spectra of phosmers CL and M are reported for comparison; the main bands that in the grafted samples strengthened due to their contribution are indicated with * (see Supplementary Material for assignments). The main bands assignable to β-sheet (β) conformation are indicated.

The IR spectra of Tussah silk fabrics grafted with phosmers are shown in Figure 10. Band wavenumbers and assignments are reported in Table S4, Supplementary Material.

IR spectra confirmed the overall β-sheet conformation of the fibers and the enrichment in ordered conformations upon grafting. Amide I, Amide II and Amide III were observed at wavenumber positions typical of β-sheet structure (in the control fabric at 1695–1622, 1511 and 1242–1222 cm^−1^, respectively), together with the spectral feature at 963 cm^−1^, characteristic of the same conformation (see Table S4, Supplementary Materials for detailed assignments [48,49,52,53]).

Upon grafting, a shift from 1622 to 1624 cm^−1^ was observed in the Amide I band, whose FWHM reduced from 51 to 46 cm^−1^ in agreement with Raman data. Similarly to *B. mori* silk fabrics, the Amide II band slightly shifted and showed an FWHM reduction (from 51 to 49 cm^−1^). The Amide I/Amide II absorbance ratio increased, thus confirming a certain rearrangement towards more regular structures. This finding was further confirmed by the decrease of the I_1242_/I_1222_ ratio (from 0.85 in control fabrics to 0.74 and 0.72 in CL and M grafted samples, respectively), identified as a marker of crystallinity [51], and by the disappearance of the 673 cm^−1^ band (not shown), attributed to unordered structures [54].

The main bands ascribable to phosmers were observed at about 1732–1717, 1454–1444, 1165–1134, 1034, 1006 and 830 cm^−1^ (see Table S4, Supplementary Materials for detailed assignments).

### 2.3. Vibrational Analyses of Grafted Wool Keratin Fabrics

**Figure 11:**
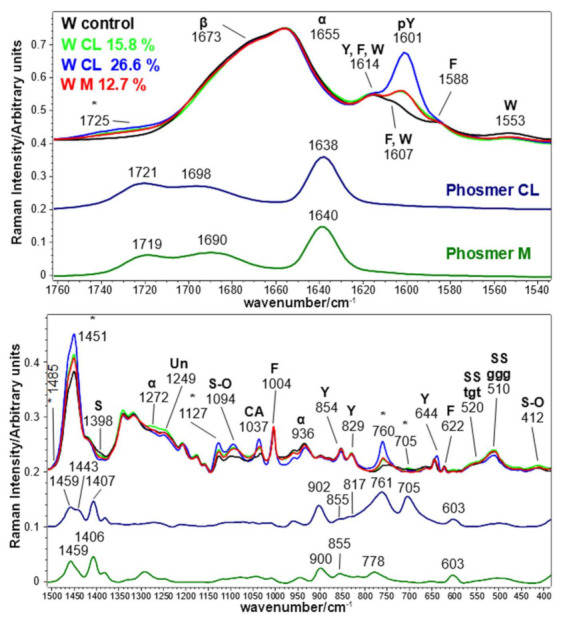
Raman spectra of wool keratin fabrics before (control) and after grafting with phosmer CL (weight gains 15.8% and 26.6%) and M (weight gain 12.7%). The spectra are normalized to the Amide I band. The spectra of phosmers CL and M are reported for comparison; the main bands that in the grafted samples strengthened due to their contribution are indicated with * (see Supplementary Material for assignments). The main bands assignable to β-sheet (β), α-helix (α) or unordered (Un) conformation as well as to tyrosine (Y), phenylalanine (F), phosphorylated tyrosine (pY), serine (S), tryptophan (W), cysteic acid (CA) and sulfated species (S-O) are indicated. The assignments to the main conformations adopted by disulfide bridges (SS), i.e., gauche-gauche-gauche (ggg) and trans-gauche-trans (tgt) are indicated, too.

Figure 11 reports the Raman spectra of control and grafted wool fabrics. Band wavenumbers and assignments are reported in Table S5, Supplementary Material. The Amide I main band at 1655 cm^−1^ and the Amide III component at 1272 cm^−1^ are typical of α-helix, i.e., the main secondary structure present in the internal cortical cells of wool, confirming that Raman spectroscopy is more sensitive to the bulk region. [55,56,57]. In addition, the band at 936 cm^−1^ is assignable to the α-helix conformation [58]. The other components at 1673 cm^−1^ (Amide I) and 1249 cm^−1^ (Amide III) are attributed to β-sheet and unordered structures, respectively. The shoulder at 1673 cm^−1^ reduced in intensity in the grafted samples; consequently, the FWHM decreased from 55 cm^−1^ to 53 cm^−1^ in CL 26.6% and M 12.7% grafted samples. The spectra of the grafted fabrics were also characterized by several bands attributed to the grafting mechanism (marked with asterisks): 1725, 1485, 1451, 1127, 760 and 705 cm^−1^ (see Table S5, Supplementary Material for detailed assignments). They were thus in similar positions compared to what was previously observed in *B. mori* silk and Tussah silk fabrics. As observed above, the spectral feature at 1127 cm^−1^ disclosed the polymerization of the phosmers onto keratin. A feature typical of wool is the presence of a pattern of disulfide bridges whose conformations can be studied through the 480–580 cm^−1^ range [42] (as detailed later, Figure S3, Supplementary Material). Under the used grafting conditions, disulphide bridges can be oxidized, and according to the literature [59,60,61], the main oxidation product detected by Raman spectroscopy appeared to be cysteic acid, as revealed by the trend of its marker band at 1037 cm^−1^ (due to sulphonate salt), which intensified in all the grafted samples. Accordingly, the area ratio between the cysteic acid marker band at 1037 cm^−1^ and the S-S band at 510 cm^−1^ increased almost linearly with phosmer content: the ratio ranged from 0.15 in the control to 0.19 (W CL 15.8%), 0.22 (W M 12.7%) and 0.42 (W CL 26.6%), indicating an increasing transformation of S-S bonds into S=O bonds. The reaction of other amino acids with persulphate cannot be excluded since strengthening the bands at 1094, 570 and 430–410 cm^−1^ (Figure 11 and Figure S3, Supplementary Material) could be ascribed to incorporating sulphate groups (see Table S5, Supplementary Material for detailed assignments [47,62,63]). The strengthening of the latter bands was observed in wool keratin after treatment with chlorosulfonic acid [47] but was not detected in hair keratin after bleaching [61].

**Figure 12:**
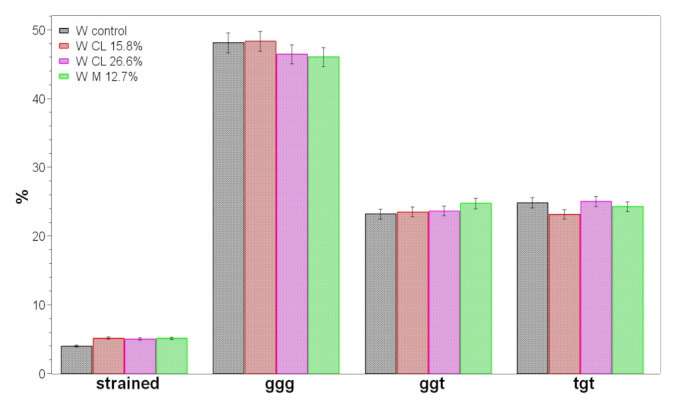
Results of the curve fitting procedure on the 580–480 cm^−1^ Raman spectral region displaying the main conformations adopted by disulfide bridges (SS), i.e., strained, gauche-gauche-gauche (ggg), gauche-gauche-trans (ggt) and trans-gauche-trans (tgt). A standard error of 5% was used for error bars [64].

The main SS stretching mode of cystine disulfide bridges was observed at 510 cm^−1^, suggesting that C_α_-C_β_-S-S-C_β_-C_α_ in wool adopt the lowest potential energy conformation, i.e., gauche-gauche-gauche [42,63,64]. Figure S3, Supplementary Material, shows detail of the SS spectral region and highlights the presence of other weaker components at 547 cm^−1^ (trans-gauche-trans), 520 cm^−1^ (gauche-gauche-trans) and 494 cm^−1^ (strained). Grafting caused an increase of the tgt (mainly in the CL 26.6% sample) and strained (mainly in the M 12.7%) components; these results were further confirmed by curve fitting analysis of the SS stretching region (Figure 12).

As observed in silk fibroins, grafting induced a reduction of the I_854_/I_829_ ratio of Tyr residues; wool keratins appeared to be more affected by the phosmers than silk fibroins. In fact, this ratio decreased from 1.27 in control to 1.00 and 0.98 in CL 15.8% and CL 26.6%, respectively, and 1.12 in the M 12.7% sample. As previously observed in fibroins, the diagnostic band of phosphorylated Tyr at 1601 cm^−1^ increased in intensity, while the δOH band of serine at 1398 cm^−1^ decreased upon grafting, suggesting an involvement of these polar amino acids in the grafting reaction. In the light of the above-reported results, it cannot be excluded that the weakening of the Ser band could also be due to its reaction with persulphate; a similar trend was observed for this band upon the reaction of wool with chlorosulphonic acid [47].

**Figure 13:**
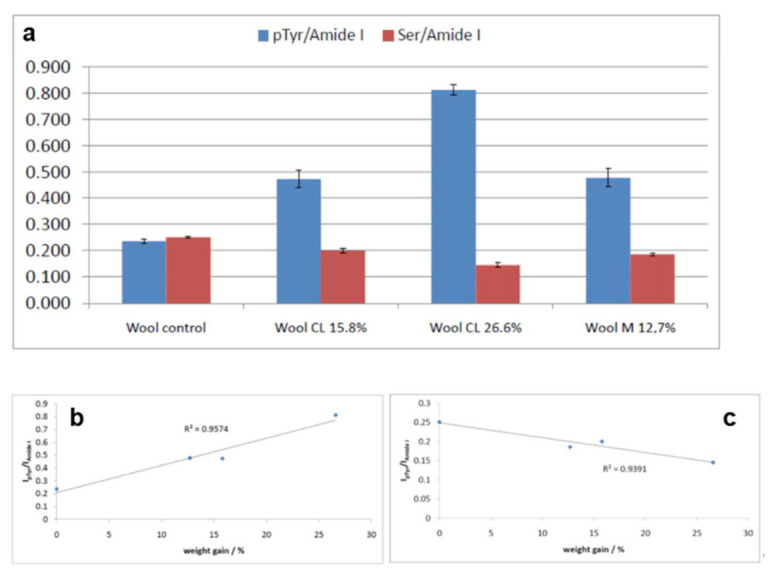
(**a**) I_pTyr_/I_Amide I_ and I_Ser_/I_Amide I_ intensity ratios (average ± standard deviation, *n* = 4) as obtained from the Raman spectra of wool fabrics under study. Trend of the I_pTyr_/I_Amide I_ (**b**) and I_Ser_/I_Amide I_ (**c**) as a function of weight gain.

Figure 13 reports the trend of the I_pTyr_/I_Amide I_ and I_Ser_/I_Amide I_ intensity ratios. Both ratios significantly changed upon grafting and were found to correlate with weight gains well. The former increased due to Tyr phosphorylation, the latter decreased due to Ser phosphorylation/sulphation.

Figure S4, Supplementary Material, shows the trend of the % changes of I_pTyr_/I_Amide I_ and I_Ser_/I_Amide I_ versus weight gain for all the fabrics under study (except for the degraded B m CL 26.5% and B m M 22.8%). As can be seen, the latter ratio decreased quite linearly with weight gain, while the former shows different trends among the different series of samples, suggesting that material-related factors determine the behavior of Tyr residues.

**Figure 14:**
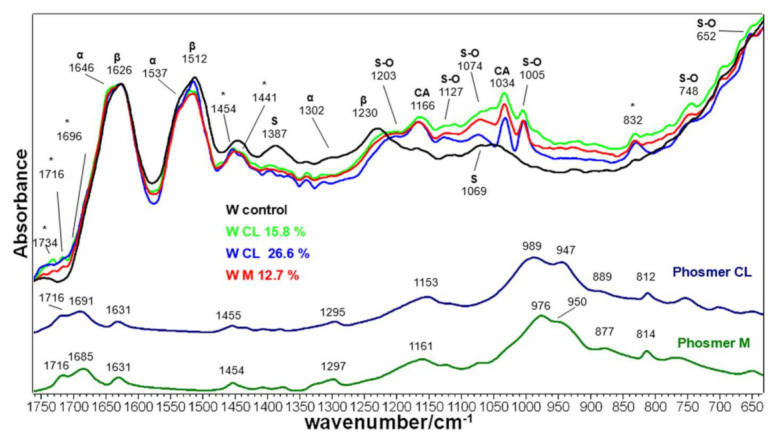
IR spectra of wool keratin fabrics before (control) and after grafting with phosmer CL (weight gains 15.8% and 26.6%) and M (weight gain 12.7%). The spectra are normalized to the Amide I band. The spectra of phosmers CL and M are reported for comparison; the main bands that in the grafted samples strengthened due to their contribution are indicated with * (see Supplementary Material for assignments). The main bands attributed to β-sheet (β) and α-helix (α) conformations, as well as to serine (S), cysteic acid (CA), and other sulfur oxidation products (S-O), are indicated.

Figure 14 shows the IR spectra of control and grafted wool fabrics. Band wavenumbers and assignments are reported in Table S6, Supplementary Material. IR spectra gave different information about the structural modification induced by grafting. In fact, IR spectroscopy in the ATR technique has been previously reported to be more sensitive to cuticle cell structure, constituting the outer layer of keratin [55,64]. Its prevailing structure appears mainly β-sheet since the main components of the Amide I, Amide II and Amide III bands are located at 1626, 1512 and 1230 cm^−1^, respectively. Specific contributions from α-helix structures typical of the cortex were the shoulder at 1646 cm^−1^ (Amide I), 1537 cm^−1^ (Amide II) and 1302 cm^−1^ (Amide III) [65]. As previously observed in Raman spectra, the contribution of β-sheet structures reduced upon grafting: the shoulder at 1646 cm^−1^ became more intense in the Amide I region (the A_1646_/A_1626_ absorbance ratio increased from 0.94 to 1.05 in grafted samples), together with the 1537 cm^−1^ shoulder in the Amide II range (the A_1537_/A_1512_ absorbance ratio increased accordingly from 0.88 to 0.95 going from control to grafted fabrics). Similarly to silk fibroins, the Amide I/Amide II absorbance ratio increased upon grafting. Contrarily to Amide I and Amide II bands, the Amide III profile was modified entirely because of the presence of the S-O vibrations typical of oxidized cysteines: the bands at 1166 and 1034 cm^−1^ are ascribable to cysteic acid (as sulphonate salt) [66,67] and confirm the above reported Raman findings. The bands at 1074 and 1127 cm^−1^ are assignable to cystine monoxide and cystine dioxide [68], respectively. All these bands were detected in the IR spectra of bleached hair [61,66]. Spectral features assignable to organic sulphate salts were detected at 1203, 1005, 748 and 652 cm^−1^ (see Table S6, Supplementary Material for detailed assignments [47]). The latter bands were observed in the IR spectra of wool fabrics treated with chlorosulphonic acid and may reveal the reaction of other amino acids with persulphate, confirming the Raman results. It cannot be excluded that SO_2_ antisymmetric stretching of sulfonamides and sulfoamines [62] could also contribute to the broad band at about 1200 cm^−1^.

New bands markers of grafting were observed at 1734, 1716, 1696, 1454, 1441 and 832 cm^−1^.

The relatively high serine content in the cuticle was confirmed by the bands at 1387 cm^−1^ (δOH) and 1069 cm^−1^ (νCO) [47]. The former reduced upon grafting/sulphation, thus confirming Raman data, while the latter overlap with the bands of phosmers and S-O oxidation products.

### 2.4. Bioactivity Tests

**Figure 15:**
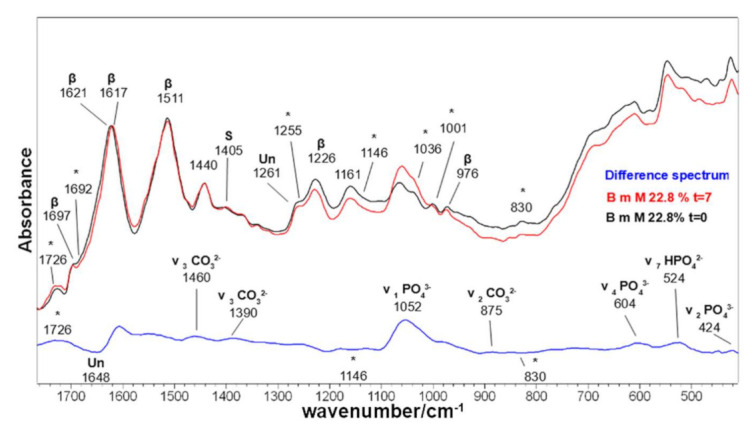
IR spectra of B m M 22.8% fabric before and after immersion in an SBF solution for 7 days. The spectra are normalized to the Amide I band. The difference spectrum was calculated by subtracting the spectrum at t = 0 from that at t = 7d. Asterisks (*) indicate the main spectral features ascribable to phosmer. The main bands assignable to β-sheet conformation (β), carbonate and phosphate are indicated.

No pH variations of the SBF solution were observed upon ageing of the samples for 7 days. Weight measurements (Table 2) showed that at 7 days, a significant weight gain occurred in most samples due to the nucleation of an inorganic phase. In silk fibroin samples, the highest weight gains were measured in fabrics grafted with the highest content of phosmer M (the B m M 22.7% and T M 12.4% samples showed weight gains of 8.5% and 4%, respectively). As an example, the IR spectrum of B m M 22.7% (i.e., the one showing the highest weight gain) is reported in Figure 15. IR spectroscopy in the ATR mode, sensitive to the first 2 microns from the surface, confirmed the hypothesis of the nucleation of a calcium phosphate phase. In fact, a significant strengthening of the band at about 1060–1040 cm^−1^ was detected due to the contribution of the ν_1_ PO_4_^3−^ stretching [69]. The A_1060_/A_Amide I_ IR absorbance ratio, identified as a marker of bioactivity, increased accordingly (Figure S5, Supplementary Material).

As can be easily seen from Figure S5, Supplementary Material, all the samples underwent a significant increase in this spectral marker, independent of the value negative or positive of the weight gain after 7 days. Actually, the sample showing the highest A_1060_/A_Amide I_ absorbance increase was wool CL 26.6% (see Figure S6, Supplementary Material), which showed a negative weight gain after 7 days. These results may suggest that in wool and silk fabrics, the nucleation of the inorganic phase is associated with a mass loss dependent on the phosmer. Raman spectra recorded on grafted samples after aging in SBF confirmed this hypothesis. As mentioned before, Raman spectroscopy is more sensitive to the bulk of the material; therefore, it appeared more suitable to follow the latter phenomenon while being less valuable to investigate the inorganic phase deposition on the surface of fabrics. Figure S7, Supplementary Material, focuses on the 1760–1580 cm^−1^ Raman range of selected grafted samples, where the weakening of the bands at 1725–1720 cm^−1^ (ester C=O of phosmer) and 1601–1599 cm^−1^ (phosphorylated Tyr) was attributed to the hydrolysis of the bonds between the phosmers and proteins during immersion in SBF. This result could indicate that part of grafted phosmer molecules is weakly bound to fabrics, i.e., those forming phosphodiester bonds with Tyr residues.

This hypothesis is further confirmed by the difference spectra shown in Figure 15 and Figure S6, Supplementary Material: negative bands at about 1717, 1150 and 830 cm^−1^ correspond to the marker bands of grafting. The same spectra allowed the calcium phosphate phase to be better characterized because of the appearance of positive bands at about 605 cm^−1^ (ν_4_ PO_4_^3−^ bending [69]), 520 cm^−1^ (ν_7_ HPO_4_^2−^ bending [69]) and 478–424 cm^−1^ (ν_2_ PO_4_^3−^ bending [69]). Other positive bands are associated with carbonate modes in the 1480‒1385 cm^−1^ range (ν_3_ CO_3_^2−^ stretching [69]) and at 875 cm^−1^ (ν_2_ CO_3_^2−^ bending [69]), thus confirming that the inorganic phase is a carbonated calcium phosphate containing HPO_4_^2−^ ions. The latter result is not surprising in relation to the short ageing times of the fabrics. It is well known that the first phases formed in mineralization processes both in vitro and in vivo are amorphous calcium phosphates containing HPO_4_^2−^ ions, which later mature, transforming into nanocrystalline apatites [70,71].

Minor structural changes were detected after SBF immersion: in *B. mori* fabrics, the Amide I band shifted from 1621 to 1617 cm^−1^, with a decrease of the 1648 cm^−1^ component, corresponding to unordered structures (see difference spectrum, Figure 15), thus suggesting that slight conformational changes occurred in silk fibroin, although the main conformation remained β-sheet. In wool fabrics (Figure S6, Supplementary Material), Amide I showed an increase in the α-helix contribution at 1643 cm^−1^ and the strengthening of the Amide II band compared to the Amide I band. This change in the Amide II/Amide I ratio has already been already reported for remineralized collagen [72] and was assigned to the interactions between collagen and Ca^2+^ ions.

**Figure 16:**
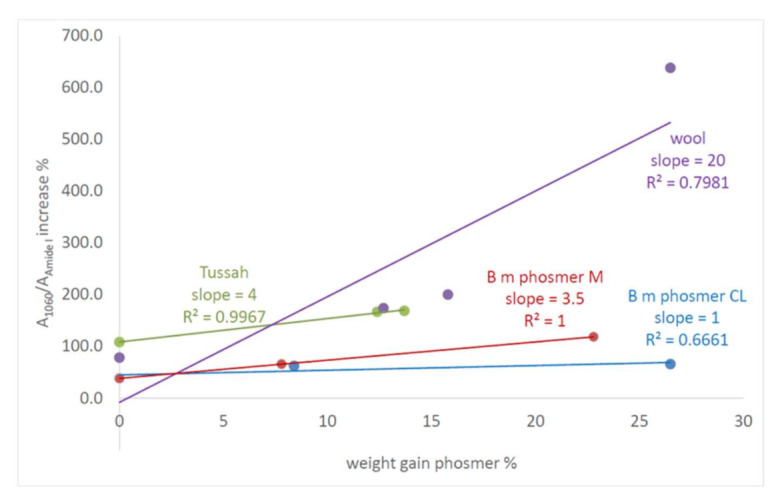
Trend of the A_1060_/A_Amide I_ IR absorbance ratio increase % after 7 days immersion in the SBF solution, as a function of the weight gain % after phosmer grafting.

Based on the reported results, the IR A_1060_/A_Amide I_ absorbance ratio appeared to be the best parameter to analyze the bioactivity of the samples. For all the samples, the % increase of this parameter well correlated with the weight gain measured after grafting experiments (Figure 16), suggesting more quantitatively that the fabrics having incorporated the highest amounts of phosmer were the most bioactive.

Good correlations were also found between the above-mentioned A_1060_/A_Amide I_ IR absorbance ratio increase % after 7 days immersion in the SBF solution and the Raman I_pTyr_/I_Amide I_ intensity ratios of the starting materials (i.e., at t = 0), for Tussah and wool fabrics (Figure S8, Supplementary Material) This result further strengthens the idea that the phosphate groups incorporated into the fibers upon grafting act as nucleating sites of the mineral phase. The trends observed in Figure 16, i.e., the significantly higher slope observed for the wool fabrics, suggest that sulphate/sulphonate groups play an important role in mineralization, in agreement with the literature [22].

## 3. Materials

### 3.1. Materials

Silk fabrics from *B. mori* and *A. pernyi* and wool fabrics were used as substrates. Phosmer M (2-phosphonooxyethyl 2-methylprop-2-enoate) and phosmer CL ((3-chloro-2-phosphonooxypropyl) 2-methylprop-2-enoate) grafting monomers (commercial names, see Table 1 for chemical formulas) are commercial products of Uni chemicals, Ltd., Osaka, Japan.

### 3.2. Grafting

The grafting reaction was undertaken according to the previously reported procedure [18]. A simplified reaction scheme between phosmers and silk fibroins is shown in Figure S2, Supplementary Material. Fabrics were immersed in a grafting system containing 1.0 g/L nonionic emulsifying agent (polyoxyethylenealkylphenylether), 3% ammonium persulfate (grafting initiator) and different amounts of phosmer (100, 120 and 200% owf). The pH of the grafting system was adjusted to 3.0 by adding 0.5 g/L formic acid solution. A material to liquor ratio of 1:30 was maintained for the course of the reaction. The grafting system was gradually heated from room temperature to 80 °C in a thermostatically controlled bath and kept at the same temperature for different times (i.e., 40′ and 90′). At the end of the reaction, the grafted fabric was soaked in a solution containing 1.0 g/L nonionic detergent at 80 °C for 30 min and rinsed with water thoroughly. The unreacted phosmer was removed via treatment with acetone at 55 °C for one h and then with 1.0 g/L nonionic emulsifying agent solution at 80 °C for 30 min. The specimens were then washed three times with hot water and air-dried at 100–105 °C for two h.

The weight gain was calculated based on the air-dried weights before and after the grafting reaction. A correction was made for the sample weight loss in the reaction system during the treatment via comparison with a blank sample treated without the monomer.

### 3.3. Bioactivity Tests

The bioactivity of control and grafted fabrics (test samples of 2 × 3 cm^2^ and 30 ± 5 mg) was assessed by immersing the samples into a simulated body fluid (SBF) buffered at pH 7.5 at 37 °C and containing the concentrations of the following ions: 142 mM Na^+^, 4 mM K^+^, 2.5 mM Ca^2+^, 148.8 mM Cl^−^, 4.2 mM HCO_3_^−^, 1 mM HPO_4_^2−^ [27]. The samples were then washed thoroughly with water, air-dried and weighted in order to calculate the % weight gain according to the equation:

% weight gain = 100 (w_7_ − w_0_)/w_0_

where w_7_ is the dry weight after 7 days of immersion in SBF and w_0_ is the initial weight.

### 3.4. Vibrational Spectroscopy

Raman spectra were recorded in triplicate using a Bruker MultiRam FT-Raman spectrometer equipped with a cooled Ge-diode detector. The excitation source was an Nd^3+^-YAG laser (1064 nm) in the backscattering (180°) configuration. The focused laser beam diameter was about 100 μm, the spectral resolution 4 cm^−1^ and the laser power at the sample about 80 mW.

The relative contents of disulfide bridges and cysteic acid (as sulfonate salt, R-SO_3_^−^) were evaluated through the Raman bands at about 510–520 cm^−1^ and 1040 cm^−1^, respectively. A_1040_/A_s-s_ was calculated, where A_s-s_ and A_1040_ were the areas of the bands assignable to disulfide bridges (calculated drawing a baseline between 482 and 585 cm^−1^) and to cysteic acid at about 1040 cm^−1^ (calculated drawing a baseline between 1070 and 1020 cm^−1^), respectively.

The 1740–1570 cm^−1^ (Amide I) and 580–470 cm^−1^ spectral ranges in wool samples were analyzed using a curve-fitting procedure to evaluate the content of secondary structures and the conformation of the C_α_-C_β_-S-S-C_β_-C_α_ linkage in cystine disulfide bridges, respectively.

A linear correction in the above-mentioned spectral ranges brought the baseline of the Raman spectra to approximately zero intensity. The frequencies of the band centers found in the fourth-derivative spectra (obtained with 13-point smoothing) were used as starting parameters for the curve-fitting procedure. The curve-fitting analysis was performed using the OPUS version 6.5 program, using the Levenberg–Marquardt algorithm. The Raman component profiles were described as a linear combination of Lorentzian and Gaussian functions. The content of α-helix, β-sheet, β-turns and unordered conformations was calculated from the area of the individually assigned bands (at about 1655, 1670, 1685 and 1640 cm^−1^, respectively) [42] and expressed as a fraction of the total area.

The contents of strained, gauche-gauche-gauche, gauche-gauche-trans and trans-gauche-trans C_α_-C_β_-S-S-C_β_-C_α_ conformations were determined from the areas of the bands at about 495, 505, 520 and 540 cm^−1^, respectively [42]. The content of each conformation was calculated from the area of the individually assigned bands and expressed as a fraction of the total area.

IR spectra were recorded in triplicate on a Bruker alpha Fourier transform FTIR spectrometer, equipped with a platinum attenuated total reflectance (ATR) single reflection diamond module and a deuterated lanthanum α-alanine doped triglycine sulfate (DLaTGS) detector; the spectral resolution was 4 cm^−1^, and the number of scans was 64 for each spectrum.

The Raman and ATR-IR spectroscopies were applied to the study of the fabrics to gain complementary information on the composition of the fibers. In fact, the former is sensitive to the sample bulk and the latter to the surface skin, i.e., to the first 2 μm of the sample.

## 4. Conclusions

In this study, three fabrics (wool keratin and silk fibroins from *B. mori* and *A. pernyi*) were successfully modified by grafting with two vinyl monomers containing phosphate (CL and M phosmers), previously used as flame retarding agents. The control and the grafted silk fabrics were characterized using IR and Raman spectroscopies to gain information on their chemical, physical and biocompatibility properties. Due to their different mechanism of interaction between light and matter, the two techniques allowed the surface (IR spectroscopy) and the bulk (Raman) of the materials to be studied. The Raman technique allowed the grafting mechanism to be understood, which involved the phosphorylation of Tyr and Ser residues, whose bands were also used to evaluate the affinity between the fabric and the grafting agent. Only small structural changes were detected in fibroin and keratin secondary structures and disulfide bonds (in keratins) upon grafting, although the treatment may have affected the amino acid composition, mainly in unordered domains. The ability of the treatment to enhance the bioactivity of fabrics was preliminarily evaluated by immersing the fabrics in an SBF solution for 7 days.

Interestingly, IR spectroscopy allowed the nucleation of carbonated calcium phosphate on all fabrics to be detected and evaluated. The content of this phase, evaluated through the A_1060_/A_AmideI_ IR ratio, was higher in the samples incorporating higher amounts of phosmers. Both phosphate and sulphate/sulphonate groups (in grafted wool) acted as nucleating sites.

These preliminary results were encouraging about a positive effect of grafting on the bioactivity properties of the fabrics because of a possible application in biomedical devices. Longer immersion times and cell proliferation studies are planned to support the present results.

## Figures and Tables

**Table 1:** Grafted samples analyzed in the present study via vibrational spectroscopy. Weight gains are indicated together with reaction times.

Phosmer	Type of Fabric and % Weight Gain	Reaction Time (min)	Sample Acronym
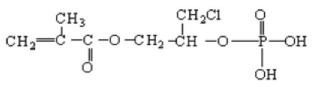	*B. mori* silk fibroin, 8.4%	40	B m CL 8.4%
	*B. mori* silk fibroin, 26.5%	90	B m CL 26.5%
	*A. pernyi* silk fibroin, 13.7%	90	T CL 13.7%
	Wool keratin, 15.8%	40	W CL 15.8%
	Wool keratin, 26.6%	90	W CL 26.6%
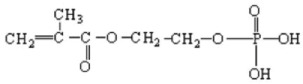	*B. mori* silk fibroin, 7.8%	40	B m M 7.8%
	*B. mori* silk fibroin, 22.8%	90	B m M 22.8%
	*A. pernyi* silk fibroin, 12.4%	90	T M 12.4%
	Wool keratin, 12.7%	40	W M 12.7%

**Table 2:** Weight gain % of the fabrics under study after 7 days in the SBF solution.

Sample	Weight Gain %
B m control	2.02
B m CL 8.4%	0.38
B m CL 26.5%	−0.64
B m M 7.8%	1.28
B m M 22.8%	8.49
T control	0.85
T CL 13.7%	1.83
T M 12.4%	4.03
W control	3.53
W CL 15.8%	2.49
W CL 26.6%	−0.39
W M 12.7%	0.43

## Data Availability

Not applicable.

## Supplementary Materials

Tables S1–S6 and Figures S1–S8: IR and Raman band assignments, additional IR and Raman data and graphics, reaction scheme.

## Sample Availability

Samples of the compounds are available from the authors.
