# Supplementary material for: Vibrational Study on Structure and Bioactivity of Protein Fibers Grafted with Phosphorylated Methacrylates
Source: Molecules. 2021 Oct 27;26(21):6487. doi: 10.3390/molecules26216487 (PMC8587459; doi:10.3390/molecules26216487)
Supplement: Supplementary file 1 [file molecules-26-06487-s001.zip › molecules-1426258-supplementary.pdf]

Supplementary Material

Vibrational study on structure and bioactivity of protein fibers grafted with phosphorylated methacrylates

Michele Di Foggia, Masuhiro Tsukada and Paola Taddei

Table S1. Main Raman band wavenumbers and assignments for *B. mori* silk fibroin fabrics before (B m control) and after grafting with phosmer CL, weight gain 26.5% (B m CL 26.5%) and phosmer M, weight gain 22.8% (B m M 22.8%). The bands having a contribution from phosmers are indicated with asterisks (assignments in bold characters).

B m control<br>ῦ / cm^{-1}	B m CL 26.5%<br>ῦ / cm^{-1}	B m M 22.8%<br>ῦ / cm^{-1}	Assignments
	1725 w	1725 w	**ester C=O stretching of phosmer [28] ***
1667 s	1667 s	1667 s	Amide I of β-sheet conformation [29,30,37]
1616 w	1615 w	1615 w	Trp, Tyr, and Phe of silk fibroin [29,30,37]
1600 w	1599 w	1601 w	Trp and Phe of silk fibroin [29,30,37] and **phosphorylated Tyr [36] ***
1555 vw	1555 vw	1555 vw	Trp of silk fibroin [29,30,37]
	1485 sh	1485 sh	**CH_2 and CH_3 bending of polymerized phosmer [33] ***
1451 m	1449 s	1451 s	CH_2 bending in silk fibroin [29,30,37] and **CH_2 and CH_3 bending of grafted phosmer [28] ***
1403 w	1403 w	1403 w	$C(CH_3)_2$ antisymmetric bending of silk fibroin [29,30,37] and Ser [41]
1368 vw	1368 vw	1368 vw	Trp of silk fibroin [29,37]
1264 m	1265 m	1266 m	Amide III of unordered conformation [29,30,37]
1229 s	1227 s	1229 s	Amide III of β-sheet conformation [29,30,37]
1171 mw	1171 mw	1171 mw	CC stretching mode of unordered conformation [29,37]
1163 mw	1163 mw	1163 mw	CC stretching mode of β-sheet conformation [29,37]
	1125 vw	1125 vw	**CC stretching of polymerized phosmer [31,32] ***
1085 s	1085 s	1085 s	CC skeletal stretching mode of β-sheet conformation [29,30,37]
1032 w	1032 w	1034 w	Phe and CC skeletal stretching of silk fibroin [29,37] and **CC stretching of polymerized phosmer [31,32] ***
1004 w	1004 w	1004 w	Phe of silk fibroin [29,30,37]
979 w	977 w	979 w	CH_3 rocking mode of β-sheet conformation [29,37]
882 w	882 w	882 w	CH_2 rocking mode of β-sheet conformation [29,37]
854 w	854 w	854 w	Tyr of silk fibroin [29,30,37] and **CH_2 in-plane bending of phosmer [32] ***
829 w	829 w	829 w	Tyr of silk fibroin [29,30,37] and **CH_2 in-plane bending of phosmer [32] ***
	810 sh	810 sh	**phosphorylated Tyr and Ser [36] ***
760 vw	760 vw	760 vw	Trp of silk fibroin [29,30] and **C-Cl and C-C stretching of phosmers [28] ***
	706 vw		**C-Cl stretching of phosmer CL [28] ***
644 w	644 w	644 w	Tyr of silk fibroin [29,37]
622 vw	622 vw	622 vw	Phe of silk fibroin [29]
	603 vw	603 w	**CCO symmetric stretching of phosmer [31,32] ***

v = very; w = weak; m = medium; s = strong; sh = shoulder.
Trp = tryptophan; Phe = phenylalanine; Tyr = tyrosine, Ser = serine.

Table S2. Main IR band wavenumbers and assignments for *B. mori* silk fibroin fabrics before (B m control) and after grafting with phosmer CL, weight gain 26.5% (B m CL 26.5%) and phosmer M, weight gain 22.8% (B m M 22.8%). The bands having a contribution from phosmers are indicated with asterisks (assignments in bold characters).

B m control<br>υ̃ / cm^{-1}	B m CL 26.5%<br>υ̃ / cm^{-1}	B m M 22.8%<br>υ̃ / cm^{-1}	Assignments
	1732 w	1726 w	**ester C=O stretching of phosmer [31,32] ***
1697 sh	1697 sh	1697 sh	Amide I of anti-parallel β-sheet conformation [44,45]
	1693 sh	1692 sh	**ester C=O stretching of phosmer [31,32] ***
1617 s	1617 s	1621 s	Amide I of β-sheet conformation [44,45]
1511 s	1513 s	1511 s	Amide II of β-sheet conformation [44,45]
	1453 sh	1454 sh	**CH_2 and CH_3 bending of phosmer [31,32] ***
1440 m	1440 m	1440 m	CH_2 bending in silk fibroin [44,45]
1405 w	1403 vw	1405 vw	Ser of silk fibroin [46,47]
1261 sh			Amide III of unordered conformation [44,45]
	1255 sh	1255 sh	**P=O stretching of phosphate group [17,43] ***
1226 m			Amide III of β-sheet conformation [44,45]
1163 m	1161 m	1161 m	CC stretching in silk fibroin [44,45]
	1152 sh	1146 sh	**COC antisymmetric stretching of phosmer [31,32] and P-O-C stretching [17,43] ***
1030 sh	1036 m	1036 m	CC stretching in silk fibroin [44,45] and **P-O stretching [17,43] ***
	999 vw	1001 vw	**P-O stretching [17,43] ***
997 vw	997 sh	997 sh	Ala-Gly sequences in silk fibroin [44,45]
976 vw	976 vw	976 vw	Ala-Gly sequences in silk fibroin [44,45]
	880 vw	877 vw	**CH_2 rocking of phosmer [31] ***
	830 vw	830 vw	**P-O-C bending [31] ***

v = very; w = weak; m = medium; s = strong; sh = shoulder.
Ser = serine.

Table S3. Main Raman band wavenumbers and assignments for Tussah silk fibroin fabrics before (T control) and after grafting with phosmer CL, weight gain 13.7% (T CL 13.7%) and phosmer M, weight gain 12.4% (T M 12.4%). The bands having a contribution from phosmers are indicated with asterisks (assignments in bold characters).

T m control $\tilde{\upsilon}$ / cm^{-1}	T CL 13.7% $\tilde{\upsilon}$ / cm^{-1}	T M 12.4% $\tilde{\upsilon}$ / cm^{-1}	Assignments
	1720 w	1720 w	**ester C=O stretching of phosmer [28] ***
1668 s	1668 s	1668 s	Amide I of β-sheet conformation [48-50]
1614 w	1614 w	1614 w	Trp, Tyr, and Phe of silk fibroin [29]
1600 w	1599 w	1599 w	Trp and Phe of silk fibroin [29] and **phosphorylated Tyr [36] ***
1555 vw	1555 vw	1552 vw	Trp of silk fibroin [29]
1452 m	1450 s	1450 s	CH_2 bending in silk fibroin [29] and **CH_2 and CH_3 bending of grafted phosmer [28] ***
1398 w	1398 w	1398 w	$C(CH_3)_2$ antisymmetric bending of silk fibroin [29] and Ser [41]
1365 vw	1365 vw	1365 vw	Trp of silk fibroin [29]
1270÷1260 sh br	1270÷1260 sh br	1270÷1260 sh br	Amide III of α-helix and unordered conformations [48-50]
1242-1230 s	1242-1230 s	1242-1230 s	Amide III of β-sheet conformation [48-50]
	1128 w	1128 w	**CC stretching of polymerized phosmer [31,32] ***
1094 s	1094 s	1094 s	Ala-rich sequences in β-sheet conformation [48-50]
1069 s	1069 s	1069 s	Ala-rich sequences in β-sheet conformation [48-50]
1032 w	1036 w	1036 w	Phe and CC skeletal stretching of silk fibroin [29] and **CC stretching of polymerized phosmer [31,32] ***
1013 w	1009 w	1009 w	Phe of silk fibroin [29] and **CC stretching of polymerized phosmer [31,32] ***
965 m	965 m	965 m	Ala-rich sequences in β-sheet conformation [48-50]
907 s	907 s	907 s	Ala-rich sequences in β-sheet conformation [48-50]
854 w	854 w	854 w	Tyr of silk fibroin [29,30,37]
829 w	829 w	829 w	Tyr of silk fibroin [29,30,37]
760 vw	761 vw	761 vw	Trp of silk fibroin [29] and **C-Cl and C-C stretching of phosmers [28] ***
644 w	644 w	644 w	Tyr of silk fibroin [29]

v = very; w = weak; m = medium; s = strong; sh = shoulder; br = broad.
Trp = tryptophan; Phe = phenylalanine; Tyr = tyrosine, Ser = serine.

Table S4. Main IR band wavenumbers and assignments for Tussah silk fibroin fabrics before (T control) and after grafting with phosmer CL, weight gain 13.7% (T CL 13.7%) and phosmer M, weight gain 12.4% (T M 12.4%). The bands having a contribution from phosmers are indicated with asterisks (assignments in bold characters).

T control<br>$\tilde{\upsilon}$ / cm^{-1}	T CL 13.7%<br>$\tilde{\upsilon}$ / cm^{-1}	T M 12.4%<br>$\tilde{\upsilon}$ / cm^{-1}	Assignments
	1732-1717 w	1732-1717 w	**ester C=O stretching of phosmer [31,32] ***
1695 sh	1695 sh	1695 sh	Amide I of anti-parallel β-sheet conformation [48]
1622 s	1622 s	1624 s	Amide I of β-sheet conformation [52]
1511 s	1513 s	1513 s	Amide II of β-sheet conformation [52]
	1453 sh	1454 sh	**CH_2 and CH_3 bending of phosmer [31,32] ***
1444 m	1444 m	1444 m	CH_2 bending in silk fibroin [52] and **CH_2 and CH_3 bending of phosmer [31,32] ***
1242-1222 m	1242-1218 m	1242-1218 m	Amide III of β-sheet conformation [48,49]
1165 m	1165-1134 m	1165-1134 m	CC stretching in silk fibroin [44,45] and **COC antisymmetric stretching of phosmer [31,32] and P-O-C stretching [17,43] ***
1030 sh	1034 mw	1034 mw	CC stretching in silk fibroin [44,45] and **P-O stretching [17,43] ***
	1006 mw	1006 mw	**P-O stretching [17,43] ***
963 mw	963 mw	963 mw	CH_3 rocking in a β-sheet conformation [49] or Ala-Ala linkages [53]
	830 w	830 w	**P-O-C bending [31] ***
673 vw			unordered conformations [53]

v = very; w = weak; m = medium; s = strong; sh = shoulder.
Ser = serine.

Table S5. Main Raman band wavenumbers and assignments for wool keratin fabrics before (W control) and after grafting with phosmer CL, weight gain 26.6% (W CL 26.6%) and phosmer M, weight gain 12.7% (W M 12.7%). The bands having a contribution from phosmers are indicated with asterisks (assignments in bold characters), those assignable to oxidation products are indicated with triangles (assignments in italic characters).

W m control $\tilde{\upsilon}$ / cm^{-1}	W CL 26.6% $\tilde{\upsilon}$ / cm^{-1}	W M 12.7% $\tilde{\upsilon}$ / cm^{-1}	Assignments
	1725 w	1725 w	**ester C=O stretching of phosmer [28] ***
	1705 sh	1705 sh	**ester C=O stretching of phosmer [28] ***
1673 sh	1673 sh	1673 sh	Amide I of β-sheet/unordered conformation [55-57]
1655 s	1655 s	1655 s	Amide I of α-helix conformation [55-57]
1614 w	1614 w	1614 w	Trp, Tyr, and Phe of wool keratin [55-57]
1607 sh	1601 s	1601 s	Trp and Phe of wool keratin [55-57] and **phosphorylated Tyr [33] ***
1588 w	1588 sh	1588 sh	Phe of wool keratin [55-57]
1553 vw	1553 vw	1553 vw	Trp of wool keratin [55-57]
	1485 sh	1485 sh	**CH_2 and CH_3 bending of polymerized phosmer [33] ***
1451 m	1451 s	1451 s	CH_2 bending in wool keratin [55-57] and **CH_2 and CH_3 bending of grafted phosmer [28] ***
1398 w	1398 vw	1398 vw	$C(CH_3)_2$ antisymmetric bending in wool keratin [55-57] and Ser [41]
1272 m	1272 m	1272 m	Amide III of α-helix conformation [55-57]
1249 m	1249 m	1249 m	Amide III of β-sheet/unordered conformation [55-57]
1127 m	1127 m	1127 m	CC stretching of wool keratin [55-57] and **CC stretching of polymerized phosmer [31,32] ***
1098 w	1094 m	1094 m	CC stretching of wool keratin [55-57] and *SO_2 symmetric stretching [47,62]* ▲
1032 w	1037 s	1037 s	Phe and CC stretching of wool keratin [55-57] and *cysteic acid [59-61]* ▲
1004 s	1004 s	1004 s	Phe of wool keratin [55-57]
936 w	934 w	934 w	α-helix conformation [58]
854 w	854 w	854 w	Tyr of wool keratin [55-57]
829 w	829 w	829 w	Tyr of wool keratin [55-57]
760 vw	760 m	760 m	Trp of wool keratin [55-57] and **C-Cl and C-C stretching of phosmers [28] ***
	705 vw		**C-Cl stretching of phosmer CL [28] ***
644 w	644 w	644 w	Tyr of wool keratin [55-57]
622 w	622 w	622 w	Phe of wool keratin [55-57]
	570 sh	570 sh	*SO_3 bending [47,62]* ▲
547 sh	547 w	547 w	SS bridges, trans-gauche-trans conformation [42,63,64]
520 sh	520 sh	520 sh	SS bridges, gauche-gauche-trans conformation [42,63,64]
510 mw	510 mw	510 mw	SS bridges, gauche-gauche-gauche conformation [42,63,64]
494 sh	494 sh	494 sh	SS bridges, strained conformation [42,63,64]
430 sh	430 w	430 w	*SO_3 rocking [47,62]* ▲
412 vw	412 w	412 w	*SO_3 rocking [47,62]* ▲

v = very; w = weak; m = medium; s = strong; sh = shoulder.
Trp = tryptophan; Phe = phenylalanine; Tyr = tyrosine, Ser = serine.

Table S6. Main IR band wavenumbers and assignments for wool keratin fabrics before (W control) and after grafting with phosmer CL, weight gain 26.6% (W CL 26.6%) and phosmer M, weight gain 12.7% (W M 12.7%). The bands having a contribution from phosmers are indicated with asterisks (assignments in bold characters), those assignable to oxidation products are indicated triangles (assignments in italic characters).

W control $\tilde{\upsilon}$ / cm^{-1}	W CL 26.6% $\tilde{\upsilon}$ / cm^{-1}	W M 12.7% $\tilde{\upsilon}$ / cm^{-1}	Assignments
	1734-1716-1696 w	1734-1716-1696 w	**ester C=O stretching of phosmer [31,32] ***
1646 sh	1646 sh	1646 sh	Amide I of α-helix conformation [65]
1626 s	1626 s	1626 s	Amide I of β-sheet conformation [55,64]
1537 sh	1537 sh	1537 sh	Amide II of α-helix conformation [65]
1512 s	1515-1519 s	1513 s	Amide II of β-sheet conformation [55,64]
1444 m	1454-1441 sh	1451 sh	CH_2 bending in wool keratin [55,64] and **CH_2 and CH_3 bending of phosmer [31,32] ***
1387 m			OH bending of Ser [47]
1302 sh			Amide III of α-helix conformation [65]
1230 m			Amide III of β-sheet conformation [55,64]
	1203 m	1203 m	*SO_2 antisymmetric stretching of alkyl and aryl sulphates [47], sulphoamines and sulphoamides [62]* ▲
1169 w	1166 ms	1166 ms	*cysteic acid [66,67]* ▲
	1127 w	1127 w	*cystine dioxide [68]* ▲
	1074 sh	1074 sh	*cystine monoxide [68]* ▲
1069 m, br			CO stretching of Ser [47]
	1034 s	1034 s	*cysteic acid [66,67]* ▲
	1003 ms	1005 ms	*CO stretching of organic sulphates [47]* ▲
	832 vw	832 vw	**P-O-C bending [31] ***
	748 vw	748 vw	*SOC symmetric stretching of organic sulphates [47]* ▲
	652 vw	652 vw	*SO_3 bending of organic sulphates [47]* ▲
v = very; w = weak; m = medium; s = strong; sh = shoulder. Ser = serine.			

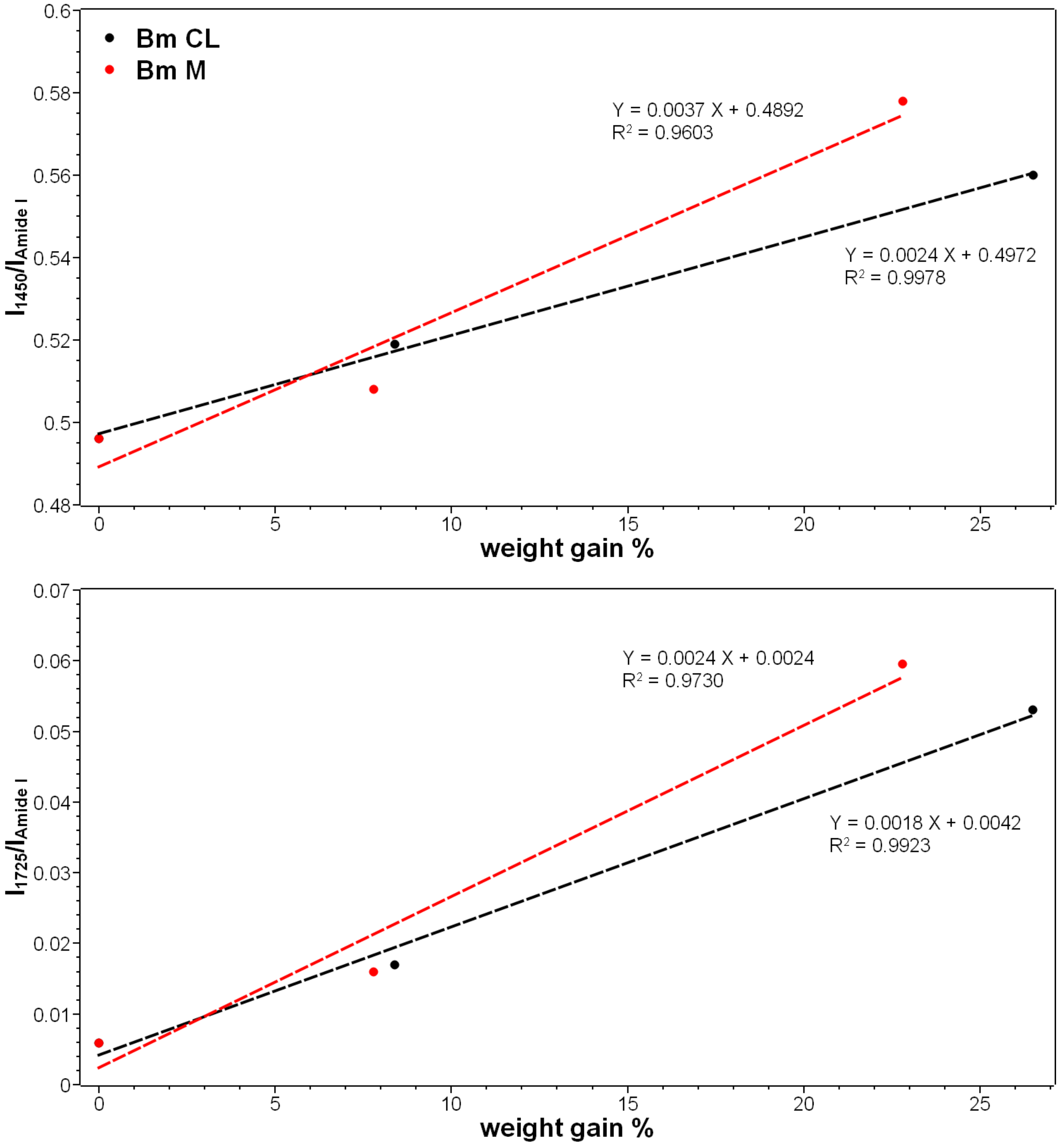

Figure S1. Trend of the $I_{1450}/I_{Amide\ I}$ and $I_{1725}/I_{Amide\ I}$ Raman intensity ratios as a function of the weight gain for the *B. mori* silk fibroin fabrics grafted with phosmer CL (black) and phosmer M (red), respectively.

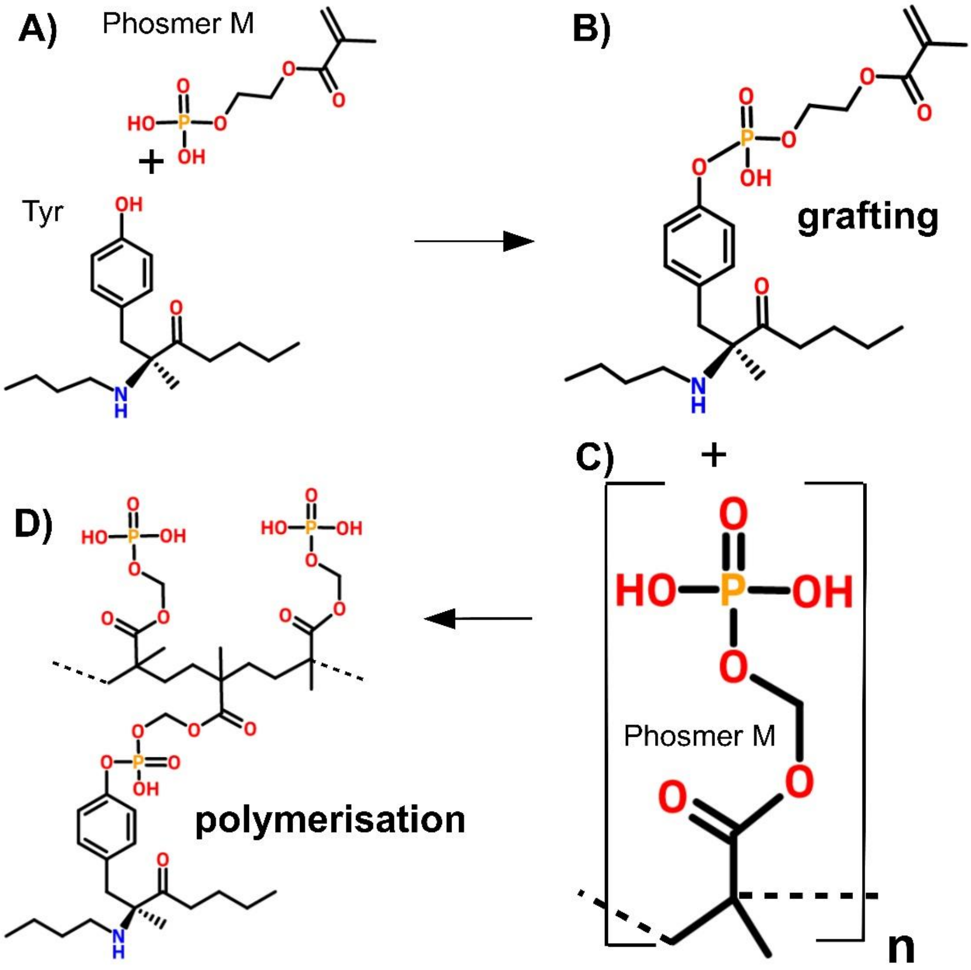

Figure S2. Reaction scheme of the functionalization of silk fibroin with phosmer M: A) Phosmer M reacts with silk fibroin (tyrosine is a preferential site); B) grafting reaction between a phosmer M molecule and the Tyr side chain of silk fibroin; C) and D) polymerization.

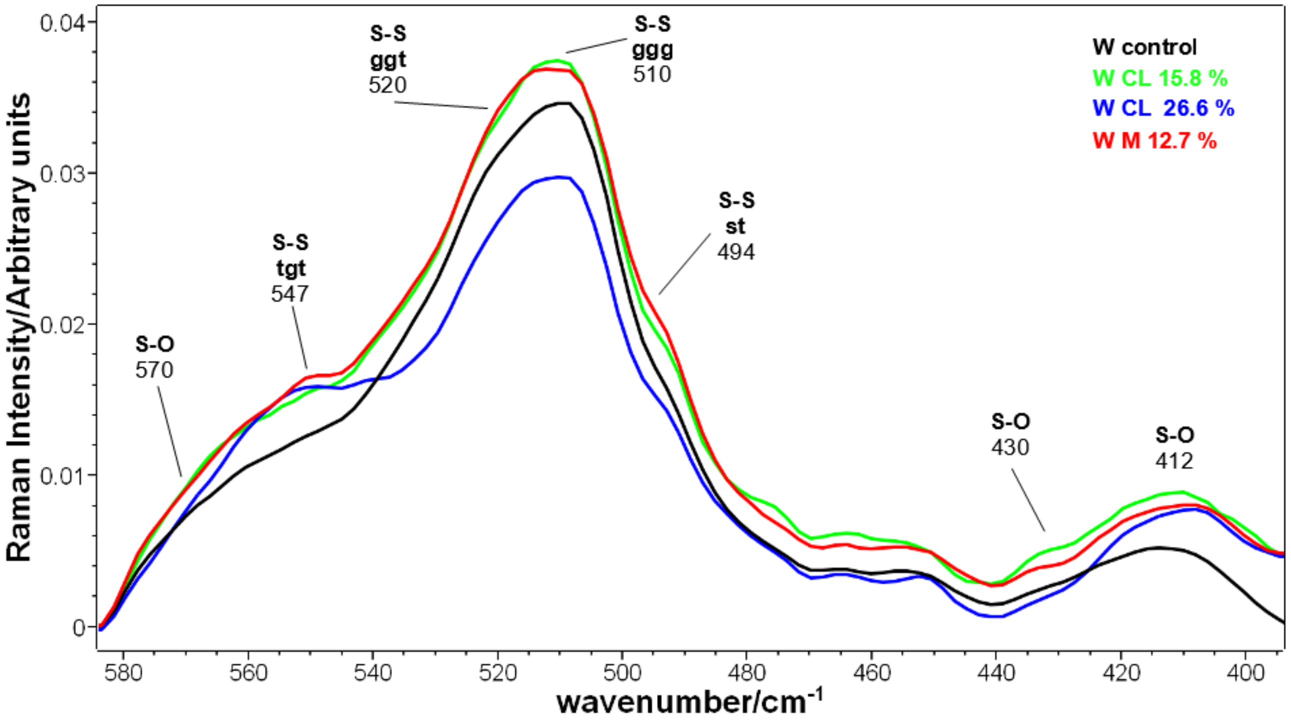

Figure S3. Raman spectra of wool keratin fabrics before (control) and after grafting with phosmer CL (weight gains 15.8% and 26.6%) and M (weight gain 12.4%). The spectra are normalized to the Amide I band. The assignments to the main conformations adopted by disulfide bridges (S-S), i.e. strained (st), gauche-gauche-gauche (ggg), gauche-gauche-trans (ggt) and trans-gauche-trans (tgt), as well to sulfated species (S-O) are indicated.

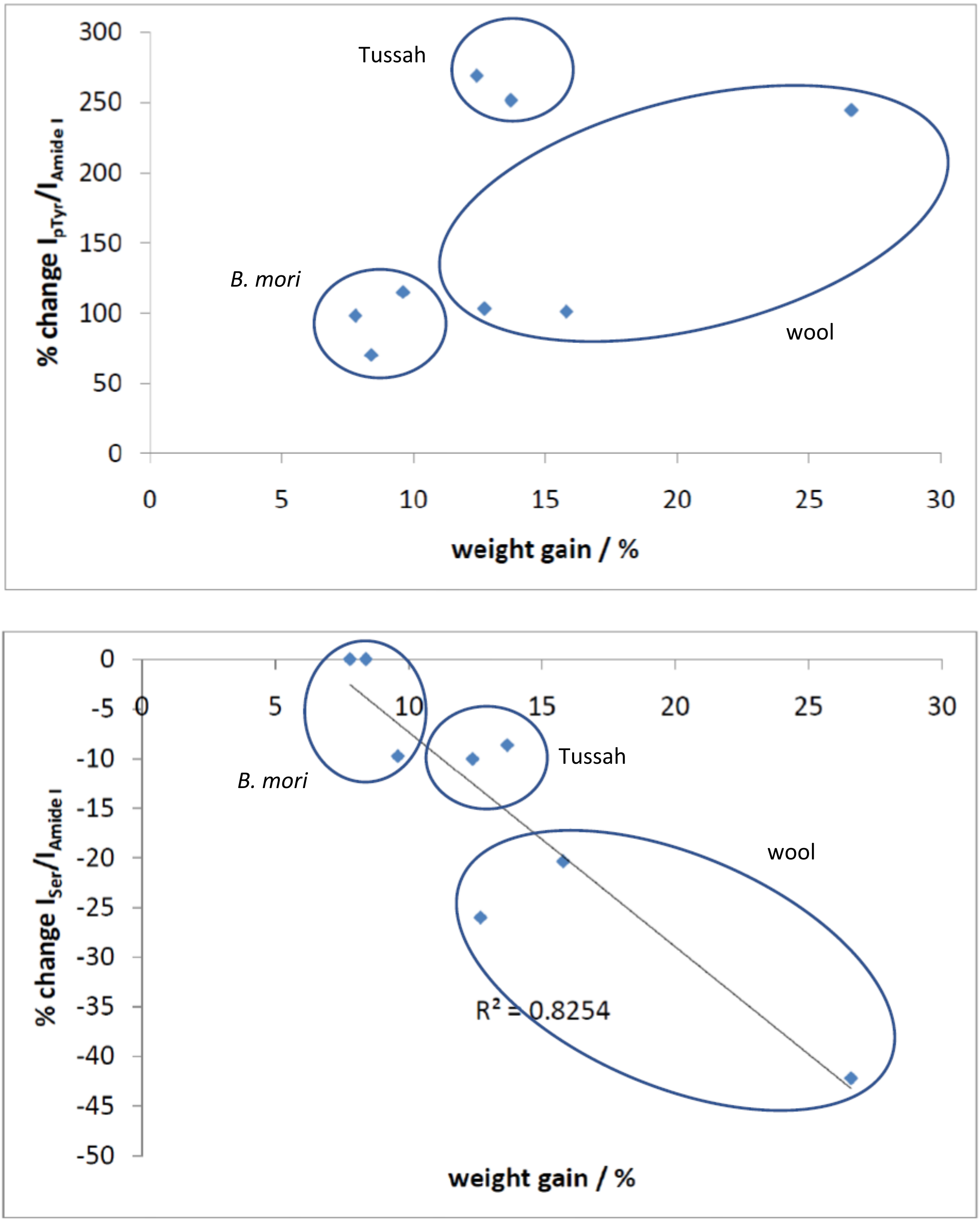

Figure S4. Trend of the % change of the $I_{pTyr}/I_{Amide\ I}$ ratio (top) and $I_{Ser}/I_{Amide\ I}$ (bottom) upon grafting as a function of weight gain for *B. mor*i (with the exception of the degraded B m CL 26.5% and B m M 22.8%), Tussah silk fibroin and wool fabrics.

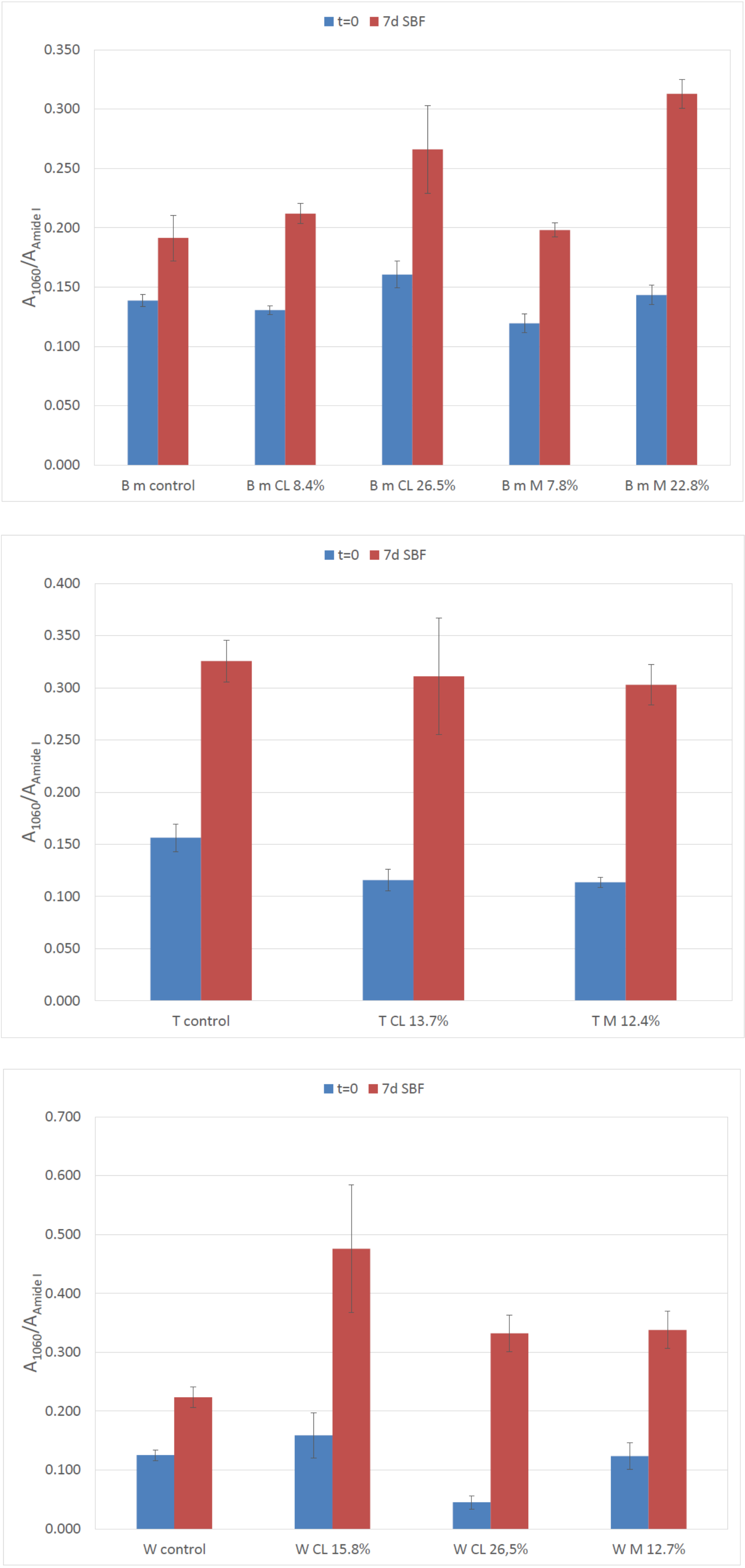

Figure S5. Values of the $A_{1060}/A_{\text{Amide I}}$ absorbance ratio (average ± standard deviation), as obtained from the IR spectra of the samples before (t=0) and after bioactivity tests (7d SBF).

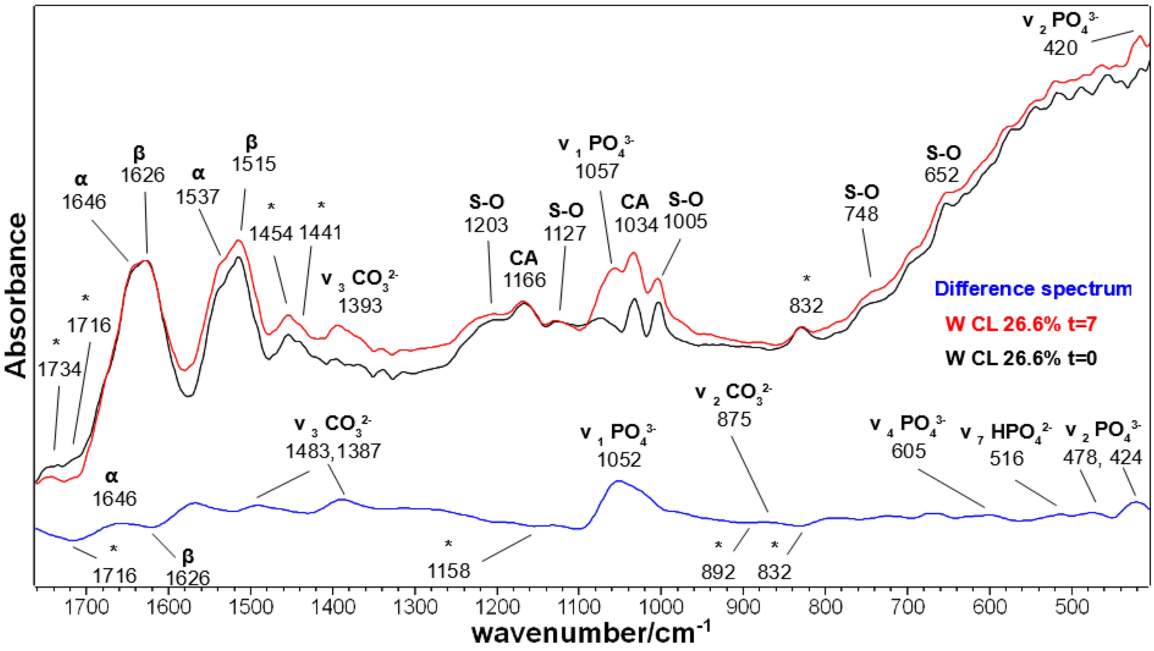

Figure S6. IR spectra of wool CL 26.6% fabric before (black) and after (red) immersion in an SBF solution for 7 days. The spectra are normalized to the Amide I band. Difference spectrum was calculated by subtracting the spectrum at t=0 from that at t = 7d. Asterisks indicate the main spectral features ascribable to phosmer. The main bands attributed to β-sheet (β) and α-helix (α) conformations, as well as to cysteic acid (CA), and other sulfur oxidation products (S-O), carbonate and phosphate are indicated.

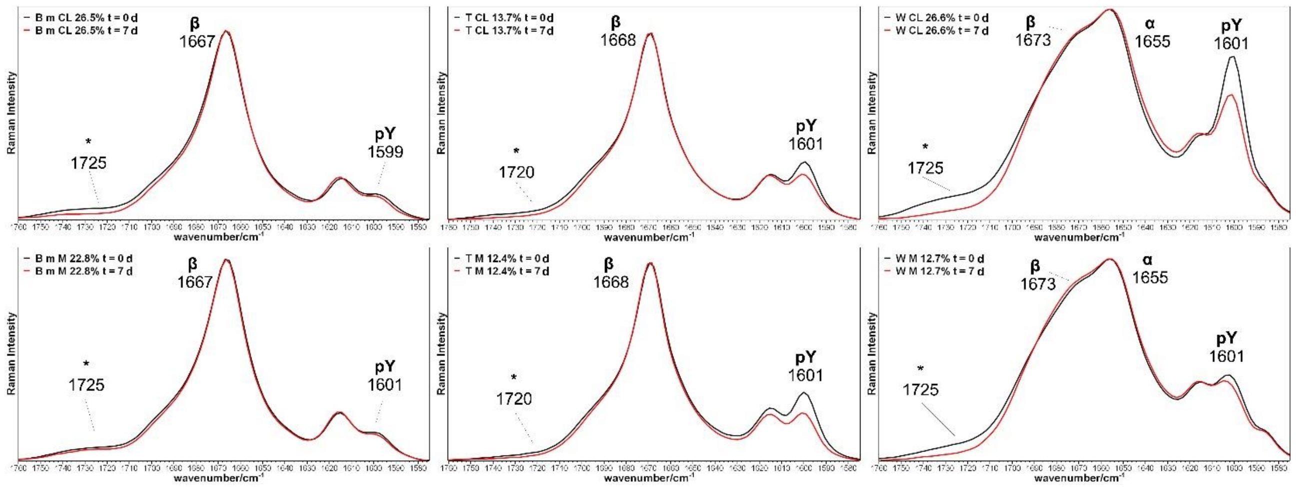

Figure S7. Raman spectra of selected grafted fabrics before (black) and after 7 days immersion in SBF (red). The spectra are normalized to the Amide I band. The main bands assignable to β-sheet (β) or α-helix (α) conformation as well as phosphorylated tyrosine (pY), are indicated.

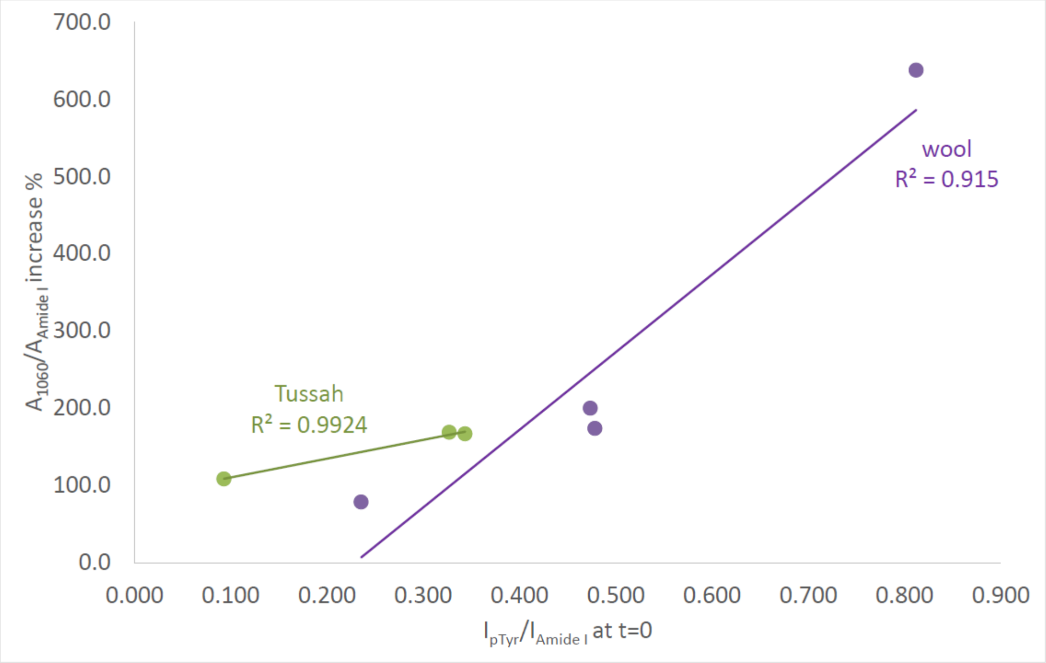

Figure S8. Trend of the $A_{1060}/A_{Amide\ I}$ IR absorbance ratio increase % after 7 days immersion in the SBF solution and the Raman $I_{pTyr}/I_{Amide\ I}$ intensity ratios of the starting materials (i.e., a t=0), for Tussah and wool fabrics.
